# Enhanced Tumor Diagnostics via Cyber-Physical Workflow: Integrating Morphology, Morphometry, and Genomic MultimodalData Analysis and Visualization in Digital Pathology

**DOI:** 10.3390/s25144465

**Published:** 2025-07-17

**Authors:** Marianna Dimitrova Kucarov, Niklolett Szakállas, Béla Molnár, Miklos Kozlovszky

**Affiliations:** 1 Doctoral School of Applied Informatics and Applied Mathematics, Óbuda University, 1034 Budapest, Hungary; 2 BioTech Research Center, Óbuda University, 1034 Budapest, Hungary; kozlovszky.miklos@nik.uni-obuda.hu; 3 3DHistech Ltd., 1141 Budapest, Hungary; szakallasn3@student.elte.hu (N.S.); bela.molnar@3dhistech.com (B.M.); 4 Department of Biological Physics, Faculty of Science, Eötvös Lóránd University, 1053 Budapest, Hungary; 5 John von Neumann Faculty of Informatics, Óbuda University, 1034 Budapest, Hungary; 6 Medical Device Research Group, LPDS, Institute for Computer Science and Control (SZTAKI), Hungarian Research Network (HUN-REN), 1111 Budapest, Hungary

**Keywords:** applied robotics, image processing of morphology and morphometry, single cell, laser microdissection, DNA sequencing, genomic mutations, database integration, fused analysis, visualization, digital pathology

## Abstract

The rapid advancement of genomic technologies has significantly transformed biomedical research and clinical applications, particularly in oncology. Identifying patient-specific genetic mutations has become a crucial tool for early cancer detection and personalized treatment strategies. Detecting tumors at the earliest possible stage provides critical insights beyond traditional tissue analysis. This paper presents a novel cyber-physical system that combines high-resolution tissue scanning, laser microdissection, next-generation sequencing, and genomic analysis to offer a comprehensive solution for early cancer detection. We describe the methodologies for scanning tissue samples, image processing of the morphology of single cells, quantifying morphometric parameters, and generating and analyzing real-time genomic metadata. Additionally, the intelligent system integrates data from open-access genomic databases for gene-specific molecular pathways and drug targets. The developed platform also includes powerful visualization tools, such as colon-specific gene filtering and heatmap generation, to provide detailed insights into genomic heterogeneity and tumor foci. The integration and visualization of multimodal single-cell genomic metadata alongside tissue morphology and morphometry offer a promising approach to precision oncology.

## 1. Introduction

The human genome consists of approximately 25,000 genes, and about 600 of them are related to tumors. These genes and their combinations can mutate up to 3.5 million ways. Within a tumor, an average of four to five different gene defects are collectively responsible for abnormal cell division. Tumor heterogeneity, which is the existence of diverse cellular populations within a single tumor, has emerged as a pivotal concept in oncology, influencing both our understanding of cancer biology and the development of treatment strategies.

The initial understanding of tumor heterogeneity dates back to the early 20th century, when pathologists first documented the variability in tumor cell morphology [1]. However, it was not until the 1970s and 1980s that more systematic studies began to explore the genetic and phenotypic diversity within tumors. Early work by researchers such as Folkman on angiogenesis [2] and Kinzler and Vogelstein on genetic mutations [3] highlighted that tumors are not monolithic entities but dynamic ecosystems with heterogeneous characteristics. The turn of the millennium brought significant advances, with the advent of high-throughput genomic technologies. The work of Garber et al. [4] and Perou et al. [5] established that tumors can be classified into distinct subtypes based on their gene expression profiles, underscoring the complexity of tumor heterogeneity at the molecular level. Subsequent studies, including those by Yachida et al. [6] and Jones et al. [7], used next-generation sequencing (NGS) to uncover extensive genetic diversity within tumors, demonstrating that tumor heterogeneity encompasses not only genetic variation but also epigenetic and phenotypic diversity. Current knowledge of tumor heterogeneity [8] reveals that it can be categorized into inter-tumor heterogeneity, which refers to differences between tumors of the same type across individuals, and intra-tumor heterogeneity, which pertains to the variability within a single tumor [9].

Tumor variability has profound implications in its interactions with the immune system, leading to variable immune responses as a consequence of heterogeneity [10]. It significantly affects interactions between immune cells and tumor cells, which are critical for disease progression and treatment outcomes. The tumor microenvironment (TME), including variations in cytokine/chemokine or cytotoxic activity, also plays a key role in shaping these immune responses that can suppress or promote tumor growth. A study by Germanà et al. (2024) highlighted that tumors often evade immune detection through immune checkpoint molecules, particularly the PD-1/PD-L1 axis, which are critical in tumor progression and response to immunotherapy [11]. High expression of PD-L1 in cancer correlates with a worse prognosis but also indicates potential therapeutic targets, with inhibitors of PD-1/PD-L1 showing promising results. These findings underscore the need for personalized immunotherapy approaches targeting specific immune cells and pathways to overcome the challenges of resistance to therapy, disease progression, and the emergence of aggressive tumor subclones [12] posed by tumor heterogeneity.

Rapid advancement of genomic technologies has significantly transformed the landscape of biomedical research and clinical applications. In recent decades, mapping and patient-specific identification of mutated genes have been utilized in clinical practice. Their purpose is to detect a tumor in its earliest possible stage based on genomics, in addition to preliminary tissue morphology. To provide more effective personalized treatment for patients, it is necessary first to understand the different types of genetic indications that cancer possesses in the patient, as well as its formation and spreading mechanisms. Without these insights, we cannot measure or intervene in cancer effectively. Hence, only drastic methods such as chemotherapy can be applied with shared success, which does not factor in the individual characteristics of a patient.

The internationally prominent leading companies in the field, such as 10X Genomics, Bruker Corporation (formerly NanoString Technologies), Oncopass, and TurbineAI, have started to explore both the macro and micro genomic research fields but from slightly different perspectives. This section provides an overview of each company by comparing its technologies, advantages, and limitations.

### 1.1. 10X Genomics

10X Genomics has emerged as a leader in single-cell genomics and spatial transcriptomics [13]. Their flagship product, the Xenium In Situ platform [14], enables researchers to perform high-throughput subcellular mapping of hundreds of RNA targets, revealing new insights into cellular structure and function. By utilizing fluorescent probe hybridization, imaging, and probe removal [15], 10X Genomics captures and processes thousands of single cells simultaneously, generating comprehensive genomic data that reveals intricate cellular landscapes. This capability allows for in-depth analysis of individual cells, which is essential in studying complex tissues and identifying rare cell populations. Furthermore, advancements in spatial analysis enable researchers to map gene expression within the tissue architecture, providing valuable insights into cellular interactions and microenvironments. However, the complexity and cost of the technology can be an obstacle for some researchers, and the vast amounts of generated data necessitate advanced computational tools and expertise, posing challenges for data interpretation and integration.

### 1.2. Bruker Corporation

The Bruker Corporation (formerly NanoString Technologies) specializes in spatial transcriptomics [16] technology. It defines an array of equipment, enabling researchers to locate transcripts, often down to the subcellular level, providing an unbiased map of RNA molecules throughout tissue sections. It employs differing combinations of microscopy, gene detection and counting, RNA sequencing, and in situ hybridization. Bruker has achievedmolecular multiplexed profiling through its nCounter, GeoMx, and CosMx [17] platforms, which, together, provide array quantification of up to 1000 RNAs or up to 100 proteins utilizing smart cyclic in situ hybridization chemistry at single-cell and subcellular resolution. This technology employs unique barcoded probes to perform in situ hybridization with RNA molecules, enabling direct quantification without the need for amplification. Bruker’s approach is particularly valuable for studies in gene expression, oncology, and immune profiling. Their products offer high specificity and sensitivity, making them suitable for low-abundance transcripts. Additionally, the straightforward workflow facilitates rapid assay development and implementation, making it accessible to a wide range of researchers. However, while nCounter allows for multiple target measurements, it is constrained by the number of probes that can be included in a single assay, which may limit comprehensive genomic profiling. The cost associated with each assay can also accumulate, particularly in large-scale studies requiring extensive multiplexing.

### 1.3. Oncompass

Oncompass Medicine, a Future Unicorn Company, has created a diagnostic platform focused on personalized oncology [18]. The platform integrates genomic data from tumor samples to generate comprehensive reports that guide clinicians in selecting targeted therapies for cancer patients. Oncompass emphasizes the clinical relevance of genomic alterations, providing actionable insights that can influence treatment decisions. This platform offers personalized recommendations based on the specific genomic profiles of tumors, enhancing the potential for effective treatment strategies. The integration of genomic information with clinical databases helps ensure that the insights apply to current treatment paradigms. However, the accuracy and relevance of recommendations are contingent on the comprehensiveness of the underlying genomic databases, which may lag behind the rapid advancements in genomics. Moreover, clinicians may require additional training to fully understand and utilize the provided genomic information.

### 1.4. TurbineAI

TurbineAI is pioneering a simulated cell model [19], using artificial intelligence to facilitate drug discovery and development processes. By analyzing vast datasets, the platform of TurbineAI predicts patient responses to therapies, identifies potential drug targets, and enhances clinical trial designs. This data-driven approach aims to streamline the drug development pipeline and improve therapeutic outcomes. The use of machine learning allows for the analysis of complex biological data, facilitating the identification of novel therapeutic targets and patient stratification. By optimizing clinical trial designs and predicting outcomes, the platform can reduce the time and cost associated with bringing new therapies to market. Nevertheless, the efficacy of machine learning models relies heavily on the quality and representativeness of the input data, which can introduce biases if not properly managed. Additionally, the complexity of AI models may pose challenges in validating predictions and translating them into clinical practice, necessitating rigorous testing and collaboration with clinical experts.

To consolidate the technological and strategic distinctions introduced above, Table 1 provides a comparative benchmarking of the core platforms developed by the industry’s most prominent players. It highlights their main products, intended functions, and key features, offering a synthesized overview of their contributions to both basic research and clinical applications.

In conclusion, each of the leading companies is continuously developing genomic diagnostic tools and services that could provide previously uncharted information on biological connections. As the field continues to advance, the integration of these innovative approaches will be crucial in enhancing our understanding of biology and improving patient care in the era of precision medicine. Our research aims to integrate the aforementioned morphological, spatial, and genomic aspects into a unified system that enables an automated, patient-specific tumor diagnostic workflow. To the best of our knowledge, existing market products that attempt to combine morphology and genomics typically analyze a smaller subset of genes (one to three disease-specific genes) across all cells within tissue layers, usually identified using gene-specific fluorescent staining techniques. In contrast, our approach challenges this model by analyzing all genes in fewer cells and visualizing them alongside morphometric data on morphology in real time. As part of this innovation, we aim to manage the resulting increased big data volume on a cell-by-cell basis, ensuring that it is accessible in a single location for more efficient analysis and interpretation. Additionally, we specifically aim to answer several key questions: Is it possible to aggregate the different types of metadata from independent pathological devices into a single system? Can we analyze and visualize morphological, morphometric, and genomic metadata together at the single-cell level? If so, what chromosomal distribution do gene mutations follow? How many genes are affected? What proportion of affected genes represent each chromosome? How do genomic mutations correlate with morphology? By addressing these questions, we aim to improve our understanding of colorectal cancer, identifying critical chromosomes as hotspots impacted during tumorigenesis.

The remaining part of the paper is organized as follows. Section 2 describes the applied methodologies of the created cyber-physical workflow, such as for scanning tissue samples, selecting morphology, calculating morphometric parameters, laser microdissection of cells, DNA sequencing of the cut cells, and analyzing the generated genomic metadata fused with open-source database information. Section 3 demonstrates the use of the implemented NGS Viewer application through a case study of a patient with colorectal cancer. The obtained results include tissue, cell, chromosome, and gene levels, as well as an aggregated evaluation of samples belonging to native and carcinoma tumor stages. Furthermore, the integration possibilities of the workflow are discussed across distinct phases, detailing the required instrumentation and reagents, estimated costs, critical configuration parameters, resulting outputs, the required technical expertise, and the associated time requirements. In Section 4, the conclusions are summarized and potential future research directions are discussed.

## 2. Materials and Methods

**Figure 1:**
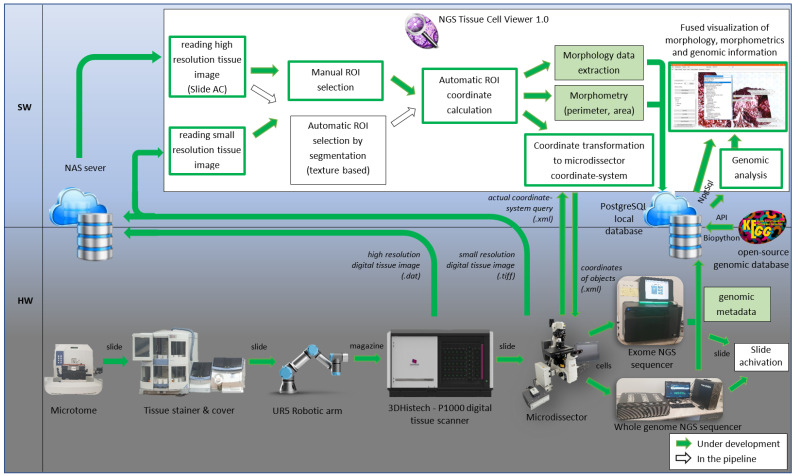
Diagram illustrating the NGS Viewer workflow, which automates the pathology process from tissue sample preparation to high-resolution scanning, followed by the identification of tissue abnormalities and automated laser microdissection of tumor-suspicious cells. These cells are amplified and sequenced for genomic analysis. The system integrates morphological, morphometrical, and genomic data, linking them with spatial coordinates to provide clinicians with a unified decision-support tool for tumor diagnostics.

We aimed to streamline the extensive pathological workflow involving many commercialized pieces of equipment, from tissue preparation through gene mutation detection of single cells, ending in the collection of gene-specific drug targets. Until now, this process has been carried out step by step, requiring the expertise and manual efforts of multiple individuals to perform the tool-specific evaluation tasks separately. We provide a solution that involves the automation of processes, with the system design plan illustrated in Figure 1. The implementation of a digital and automated pathology workflow starts with the preparation of anonymized (written informed consent S1) tissue samples on membrane slides, their robotic high-throughput and high-resolution digital scanning [20], and the subsequent determination of their tissue abnormalities [21]. Then, it continues with the automated laser microdissection of these micrometer-sized tumor-suspicious cells and cell clusters, followed by the amplification of the excised cells and DNA sequencing. Previously, metadata from each step of the workflow could only be handled and examined separately. To smooth the workflow, we set the goal to integrate them into a common platform named Next Generation Sequencing (NGS) Viewer, which saves the created morphological, calculated morphometrical, and extracted genomic metadata from NGS with the collected open-source genomic data through the whole workflow. The workflow then analyzes them together and links the extracted information to cell coordinates. This tool provides physicians with a compact visual and fused analytical framework for tumor diagnostics as a decision-support framework. To ensure reproducibility, each primary step of the workflow is described in a dedicated subsection.

### 2.1. Tissue Preparation on Membrane Slides

Biopsy samples were collected from three types of colon tissue: normal, native, and tumor tissue. The normal samples were obtained from healthy individuals and served as true non-pathological controls. The native samples (also referred to as “nat” in the literature) refer to tumor-associated normal tissue, i.e., histologically non-malignant tissue that is untreated. These regions are tumor-adjacent and considered part of the tumor microenvironment. They do not show overt malignancy but may exhibit early molecular or morphological alterations indicative of a pre-neoplastic state. Finally, the tumor samples correspond to histologically confirmed cancerous tissues in the carcinoma state used throughout the study. The distinction between “normal”, “native”, and “carcinoma” tissues is based on standard histopathological criteria, including cellular morphology, architectural organization, and the presence of dysplasia, as assessed by experienced pathologists. Furthermore, we acknowledge the potential influence of field cancerization effects, where molecular changes may extend beyond visible tumor boundaries.

The samples from the first group target the tissue layers of the tunica mucosa, tunica submucosa, and tunica muscularis, while the tumor samples were taken with the goal of capturing consecutive sections from the center of the tumor, without focusing on a specific region of interest.

The biopsied tissues were FFPE. The paraffin blocks were sectioned into 16 μm thin slices using a microtome. Instead of the classical glass slides, slices were attached to membrane slides (membrane stretched on a metal frame), since they can also be used for DNA sequencing. Depending on the diameter of the section, 2–3 adjacent slices were placed on individual membrane slides (see Figure 3). This approach ensures that if one section experiences issues such as smearing due to cutting or staining/scanning noise, the adjacent sections can still provide reliable morphological tracking.

Tissue staining was carried out in a Gemini automated stainer [22] manufactured by Thermo Fisher Scientific. During the staining process, the tissue sections were deparaffinized using >98% xylene, with xylene removal and fixation carried out by sequential immersion in 100%, 95%, and 75% anhydrous ethanol. Tissue rehydration was performed using RNase-free deionized water (ddH_2_O). For staining purposes, the classical Hematoxylin–Eosin (H&E) [23] procedure was applied with modified Harris hematoxylin (7 g/L, mercury-free) to stain the nuclei. Hematoxylin residues were thoroughly rinsed with warm (30 °C) ddH_2_O, while a 2% water-soluble eosin stain was used to enhance contrast. The eosin was rinsed with cold ddH_2_O. The stained tissue sections were cover-slipped using the ClearVue system [24] produced by the Epredia company, which was integrated into a closed-loop setup with the staining apparatus.

### 2.2. Slide Detection and Manipulation

The preparation of stained and covered slides was autonomously examined using a UR5 robotic arm (Universal Robots USA, Novi, MI, USA) [25]. For this task, a custom gripper was developed, which included a complementary metal oxide semiconductor (CMOS) camera. The detection was implemented with computer vision and image processing in real time in a dynamic environment. During the process, the robot detected black magazines storing the stained and covered slides through the darkened Plexiglas door of the ClearVue device at regular intervals. If a completed batch was found, the robot opened the door, removed the magazine using its gripper, and placed it in its temporary storage for identification of the slides within the magazine. Furthermore, the robot also closed the ClearVue door to ensure that the contamination-free staining process could continue. When the software of the tissue scanner indicated idle time to the robotic control system, the robot immediately retrieved the next detected transparent slide from the magazine in its storage and fed it into the scanner. Through a designated entry on the side of the scanner, the robot moved into the scanner while holding the given slide, placing it in the required position and at the orientation demanded by the device. Thus, the slide was forwarded to the next robotic arm within the scanner for further processing. Once the scanning of a slide was completed, the robot retrieved it from the scanner via the same channel and replaced it in its original location within the magazine. Upon completion of all samples in a magazine, the robot placed the entire magazine into the local archive. These laboratory tasks were performed asynchronously relative to the tissue staining, covering, and scanning operations, thereby achieving optimal time utilization. Further details about the functionalities, architecture, and parameters of the robotic arm working as a laboratory technician can be found in our previous works [26,27,28].

### 2.3. Scanning Slides

During the selection of the digital tissue scanner, it was only possible to choose equipment that does not damage the membrane during its scanning, which can be accomplished with the 3DHistech Panoramic P1000 scanner used in this project [29]. The P1000 also has Diagnosis (DX) and Research (RX) subtypes. Since the parameters of the membrane slide are different from those of the glass slide, most of the adjustable options, such as focus, color balancing, scanning mode, magnification, immersion type, multilayer mode, and stitching, had to be individually adjusted. Since RX is the only machine capable of these adjustments, it was chosen for this research. The experimental scanning protocol was exported. This collection of settings can be found in scanning profile S2 in the Supplementary Files and can be imported into the P1000 during the next scan. It is noted that profiles may differ between different P1000 scanners, as the hardware of the machines may differ, e.g., different cameras and different filters installed depending on the type. Additionally, the slides can be uneven; for example, the frame may be bent, and the membrane may be stretched during tissue preparation, or it might sink under the weight of the tissue. For these reasons, the attached profile is a guideline for which further manual fine-tuning per slide is advisable.

### 2.4. Image Processing of Scanned Morphology

For morphological analysis of the scanned tissue samples, algorithmic and manual choices are available in NGS Viewer. The algorithmic analysis offers a choice of classical segmentation algorithms that can detect nuclei, glands, and surface epitheliabased on morphology, as demonstrated in our previous work [21]. These yield robust results, mainly on healthy tissue samples. Specifically, the K-means algorithm was employed for cell detection, the Density-based Hysteresis Snail (DBHS) algorithm for gland detection, and the Surface Nucleus Chain-based Algorithm (SNCA) for identification of surface epithelia. The empirically optimized parameters for these algorithms are provided in Table 2. These parameters can be individually adjusted using sliders on the NGS Viewer graphical interface, allowing for fine-tuning of the segmentation.

For the morphological analysis of native and carcinoma tissue samples, manual analysis was used, involving laboratory physicians and pathologists from the field. Selected areas to be examined were sampled using standard geometric shapes (e.g., rectangles, squares, circles, ellipses, and lines) that are compatible with the microdissector system. These selected regions of interest (ROIs) were then automatically saved to the underlying database for downstream processing.

It should be emphasized that before the ROIs determined by algorithmic or manual selection can be saved, reference points must be recorded on the slide. The reason for this is that the ROI coordinates to be selected can be automatically transformed into the mm-based coordinate system of the microdissector for laser cuts. One can find the steps of reference selection in our previous work [30].

### 2.5. Morphometry Calculation

During morphometry calculations, it is integral to note that the pixel-based digital image taken with the P1000 scanner does not have the same resolution on the x- and y-axes, even though it is at the same magnification. In other words, the width and height of a pixel in the image refer to different micrometer measurements in reality. This is due to the way the digital image is produced because the scanner uses different mechanisms to move the slide-holding stage in both directions (stepper motor and chain), which means different step sizes and precision. For this reason, it was necessary to look for formulas for calculating the area and perimeter of the selected shapes, where the two axes can be handled separately. Although this does not cause problems for lines and rectangles/squares, the classic perimeter and area formulas cannot be used for circular shapes. To resolve this, a circle is treated as a special ellipse. Nevertheless, an ellipse does not have closed perimeter and area formulas by default, so it was important to use an approximation where the conversions of the two axes can be separated. Otherwise, handling the two axes together could generate substantial value discrepancies at such small dimensions. The implemented specific formulas can be seen in our previous work [31].

### 2.6. Laser Microdissection

Following the selection of ROIs from tissue samples based on morphological analysis, their laser separation and extraction from the tissue sample were performed using a CellCut (Molecular Machines & Industries (MMI), Eching, Germany) [32] laser microdissector device manufactured by Molecular Machines & Industries Ltd. (MMI). During this process, a procedure developed in the previous phase of the research was applied to automatically laser cut μm-sized areas based on a pre-evaluated, high-resolution, static image instead of manually selecting and cutting them out of a small live image. The process and execution of the method are described in detail in [30]. The dissection parameters for the procedure were empirically set with the following values: cut velocity = 30 μm/s, laser focus = 533 μm, laser power = 80% of 10 mW, cutting repeats = 2, Z drill = 0.1 μm, and focus check = 1 s.

### 2.7. DNA Sequencing

The laser microdissection and the whole-exome/genome sequencing of the tissue cells were connected through a decoding database to precisely identify the morphological positions of the corresponding mutation data in later steps. After the laser excision of cells, genomic sequencing was performed by a NextSeq 500 (Illumina, Inc., San Diego, CA, USA) [33] instrument manufactured by Illumina, which is suitable for sequencing DNA exomes, and by a PromethION Nanopore [34] machine manufactured by Oxford, which is capable of sequencing the whole genome of DNA and RNA as well.

DNA extraction and whole-genome amplification were carried out using a REPLI-g Single Cell Kit (Qiagen GmbH, San Diego, CA, USA), while whole-exome capture was completed using a QIASeq Human Exome Kit (Qiagen GmbH) according to the manufacturer’s protocol. For variant calling, the BaseSpace platform was utilized, which employs the DRAGEN pipeline [35] with the following small-variant hard-filtering thresholds [36]:

- DRAGENSnpHardQUAL: For SNP variants, if the QUAL score is below 10.41, the variant is filtered out.
- DRAGENIndelHardQUAL: For INDEL variants, if the QUAL score is below 7.83, the variant is filtered out.
- LowDepth: Variants with a depth (DP) less than or equal to 1 (DP ≤ 1) are filtered out.
- PloidyConflict: Variants whose genotypic calls are inconsistent with the chromosome ploidy are filtered out.
- base_quality: Variants are filtered if the median base quality of alternative reads at the locus falls below a threshold.
- lod_fstar: For mitochondrial contigs, if the LOD (log-likelihood) score does not exceed the threshold of 6.3, the variant is filtered out.

QUAL represents the Phred-scaled probability that the site is not a variant, computed as follows: QUAL=−10·log10 (posterior genotype probability for GT = 0/0). These filtering thresholds ensure that only high-confidence variants are considered for further analysis.

The genomic metadata per ROI obtained from the sequencing equipment were converted to .txt extension files containing around 150 different parameters of the mutations of the ROI in the form of rows, such as the HUGO symbol of the mutated gene, the location of the mutation, or the type of the variant relative to the Hg38 Human Reference Genome.

### 2.8. Genomic Analysis

**Figure 2:**
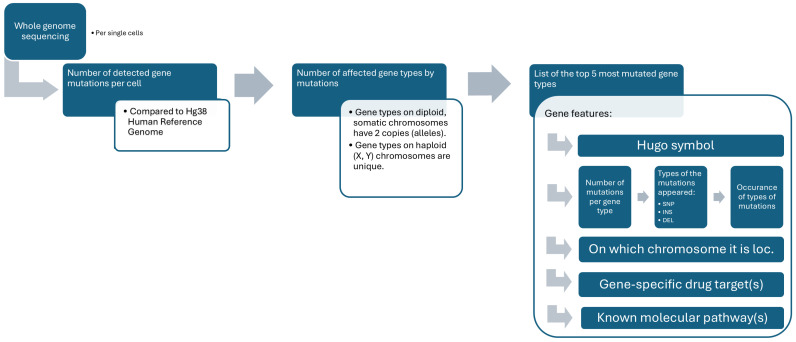
Merged genomic metadata from sequenced ROIs and the KEGG database, providing gene-level analysis for individual cells.

DNA sequencing was followed by the fitting of contigs, data cleaning, and automatic insertion of the raw genomic metadata into the fused PostgreSQL database (DB) [37]. The imported genomic metadata of a sequenced ROI was joined with the genomic data available from KEGG open databases [38] and queried from the database [39]. From this merge, the genomic information shown in Figure 2 was determined per single cell.

For an aggregated analysis of single-cell data, Table 3 was constructed, containing the genomic evaluation of each ROI at the chromosome level and collectively for tumor stage levels.

Under the unique identifier (ROI ID) of a given cell, the number of gene mutations detected for that cell per chromosome type is listed in the “Total Gene Mutations” column. Since a gene can be damaged several times, the number of different types of genes affected by these gene mutations is stored in the “Affected Distinct Genes” column. Although a gene is found on only one chromosome (type), there are two copies of the 1–21 chromosomes per cell, so different types of genes also occur twice per cell. The exception to this is the sex-linked genes on chromosomes X and Y in males because there is only one of each of these chromosomes per cell. Thus, the mutations detected in them and the genes they affect can be confidently linked to a single chromosome. However, sequencing equipment is currently not able to determine which of the given diploid, somatic chromosome pairs has the particular type of damaged gene. It can only determine the chromosome type in which the damaged gene is located. Thus, the above column can only determine distinct types of genes, not the true number of genes actually affected. Here, we can use an approximation, for example, by multiplying the number of genes by two (or dividing the number of mutations by two), assuming that diploid pairs have the same probability of being damaged by mutations.

In order to compare the amount of damaged genes calculated as described above per chromosome, it is necessary to normalize them, since chromosome sizes also vary [40]. While the size of a given chromosome cannot be fixed due to variability in human races and alleles, the gene quantities of chromosomes measured by UNCN [41] for the average human reference genome can be used as an approximation. This information is collected in the “Approximate Gene Number of Chromosome” column. If twice the number of genes is detected, different types of damaged genes (assumed) are divided by twice the gene size of the approximate chromosome (since two identical chromosomes are present), and we obtain the proportion of damaged genes in chromosomes in the “Proportion of Mutated Genes” column. This allows us to compare which chromosomes are the most damaged. Furthermore, the “Approximate Mean of Mutations/Gene” column shows how many times the genes were mutated, on average, in the damaged proportion of the chromosomes, where the total detected mutations (as they are distributed on two identical chromosomes) are divided by twice the number of affected distinct genes (assumed). Hence, the degree of gene damage can also be examined.

After the cell-by-cell evaluation, the single-cell results were aggregated for the normal, native, and carcinoma tumor phases. During this process, the proportion of mutated genes per chromosome and their degree of damage were determined for each tumor state. The tumor-stage labeling of cell samples cannot be validated, as it is based on the pathologist’s experience and subjective opinion. Along these lines, median operators were used instead of average ones for the aggregation because the mean would be heavily biased by the parameters of mislabeled cells from other classes, whereas the median avoids this.

### 2.9. Visualization of Fused Data

Tissue Cell NGS Viewer was developed by us to unite the morphological, morphometric, and genomic information extracted in the manner described above, as well as to jointly visualize this information for cells originating from different regions of the tumor. After storing this metadata in a common PostgreSQL database, the goal was to display it together within a shared graphical user interface (GUI) of NGS Viewer. Since a large amount of data needed to be placed in a relatively small area, two main display tools were used for its design, annotation, and heat mapping.

#### 2.9.1. Annotation

Due to the connection of genetic metadata and morphological coordinates in the database, the annotations could be placed directly above the ROI shapes. In order to minimize the obscuring of the morphological environment of the ROI, the annotations were formulated as drop-down and scrollable lists. They contain the evaluation results of a given ROI described in Section 2.8 that are retrieved in real time from the database with NpgSQL [42] and listed in batches. This allows for a more detailed observation of tumor heterogeneity.

#### 2.9.2. Heatmap

Meanwhile, a heatmap is introduced in the annotated areas as well, showing how much the sequenced cell (cluster) of the given annotation has mutated, thereby representing the severity of the analyzed gene mutations. Initially, all ROI annotations are transparently “colored”, since we have no information about the mutation status before sequencing the DNA. When the genomic metadata is imported into the DB, the genomic evaluation values are used to generate the following quotient to calculate a relative mutation value:

(1) $$C_{rel}^{\prime }=\frac{\sum_{i=1}^{n}m_{i}-\sum_{i=1}^{n}g_{i}}{\sum_{i=1}^{n}m_{i}},$$

where $C_{rel}^{\prime }$ is the relative cell mutation, *i* is the type of chromosome, n=[1,2,3,…,21,X,Y], $m_{i}$ is the total number of gene mutations on the *i*-th chromosome of the cell, and $g_{i}$ is the number of genes affected by the mutations on the *i*-th chromosome of the cell. In all cases, this derived value can take a value in the range of [0–1]. It takes a value of zero if all the registered gene mutations belong to different genes. At the other extreme, it takes a value of one if all of its gene mutations occur on one gene (in a tumor that can also be detected by morphology, usually 4–5 genes are mutated in higher amounts).

The calculated relative mutation value is mapped on the color scale, starting from green (0), through yellow (∼0.5), and extending to red (1). Therefore, the more gene-specifically (fewer genes with more mutations) an area has mutated, the more red it becomes. This relative mutation value makes it possible to compare annotations within a tissue sample with each other, as well as with other tissue samples, as it provides normalized values. Thus, the heatmap serves as a visual aide in identifying tumor foci.

## 3. Results

The result of our research is the Tissue Cell NGS Viewer, which fuses all the corresponding metadata of the pathological steps presented in Section 2 into a single platform. It integrates morphology, morphometry, and genomics at the cellular level, which is of immense value to diagnosticians, allowing them to focus solely on making treatment decisions. Throughout the Results section, we present the utilization of the built workflow and the developed Tissue Cell NGS Viewer application with a patient sample. We include a more in-depth explanation of different features in Supplementary Material S3.

### 3.1. System Feasibility

Insight into the operation of the integrated hardware system, which was also automated by us, as presented in previous works [26,27] during its robotic functioning can be gained from the S4 video provided in the Supplementary Files. The operation of the NGS Viewer platform, developed for the integration and processing of metadata extracted during the workflow steps, and its use by the end-user are presented in the Section 2.3, Section 2.4, Section 2.5, Section 2.6, Section 2.7, Section 2.8, Section 2.9, Section 3.1, Section 3.2, Section 3.3, Section 3.4, Section 3.5, Section 3.6, Section 3.7, Section 3.8, Section 3.9, Section 3.10 and Section 3.11. Screenshots of the NGS graphical interface provide original visual data and proof of use. Furthermore, the computational resources and requirements necessary for the real-time analysis and visualization as part of the system operation are detailed in Table 4.

### 3.2. Scanned Membrane Slides

**Figure 3:**
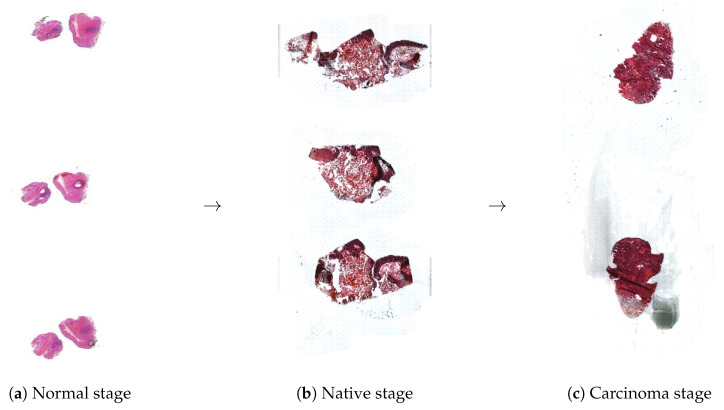
Digital slide previews of normal, native, and carcinoma biopsies digitized using a high-resolution tissue scanner. The previews, displayed in the NGS Viewer, include a thumbnail of the full slide and a high-resolution subpart for detailed examination.

Biopsied tissue samples were digitized with a Pannoramic 1000 scanner (3DHISTECH, Budapest, Hungary), which provides a high-resolution digital image stored as data in its own Mirax Digital Slide (MRXS) format. An image is not a single large file but a directory containing several files with a DataXXXX.dat extension and one file with an Index.dat extension. The whole-slide MRXS-type digital images converted to TIFF format (S5–S7) are available in the Supplementary Files. The scanned normal, native, and carcinoma digital tissue previews are presented in Figure 3a–c. Once a slide is loaded into NGS Viewer, several processing options become active. In the Viewer, one can click and zoom anywhere on the preview images above to navigate to the desired part of the tissue that they wish to examine.

### 3.3. Algorithmic Selection

**Figure 4:**
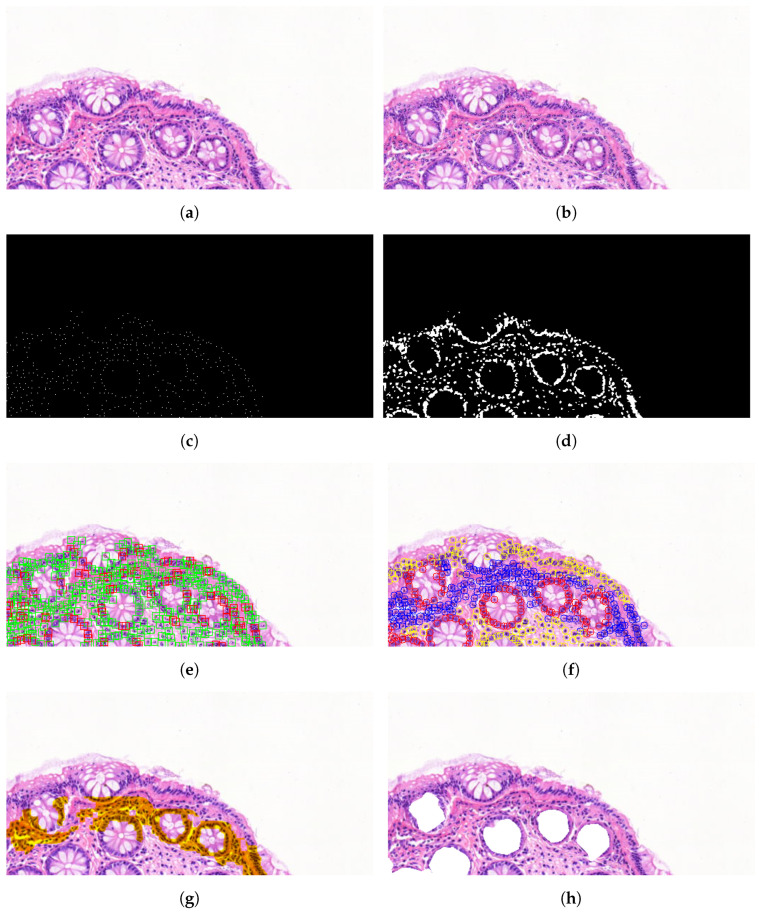
Steps of the algorithmic segmentation and classification pipeline. (**a**) Original region of interest selected for morphology analysis (Sample “G”); (**b**) detected nuclei marked in white, overlaid on the original tissue region; (**c**) binary representation of the detected nuclei, shown without background to visualize density and spatial distribution; (**d**) boundary delineation of full cell areas based on detected nuclei; (**e**) quality assessment of the delineated cells, with cells falling within the normal morphological parameter range marked in green, whereas those deviating from it are marked in red; (**f**) tissue-level classification of cells based on their associated tissue region, with stromal cells marked in yellow, gland-associated cells in red, and cells aligned along the epithelium in blue; (**g**) identification of the adjacent surface epithelial region based on the classified cells, aiding in the localization of the tissue sample’s boundary and outer edge; (**h**) detection and masking of glands inferred from the spatial clustering of cells classified as glandular.

In case of algorithmic analysis, the segmentation algorithms for nuclei, cells, glands, and surface epithelia—along with their parameters—can be configured and fine-tuned as described in Section 2.4. The processing and evaluation steps of this pipeline are illustrated with a representative sample region in Figure 4.

Panel (a) shows a selected region of interest extracted from the scanned digital tissue. In (b), the detected nuclei are overlaid in white on the original image to retain spatial context. The corresponding binary mask of these nuclei, shown in (c), highlights their density and spatial distribution, independent of tissue background. Full-cell regions are delineated in (d) by expanding from the nucleus boundaries using morphological constraints. Panel (e) presents a quality assessment of the segmented cells based on morphological criteria: cells within normal ranges are marked in green, while abnormal ones are marked in red. The classified cells in (f) are color-coded by tissue association: stromal (yellow), glandular (red), and epithelium-aligned (blue). Based on this classification, (g) shows the inferred surface epithelium region, while (h) displays the detected and masked gland areas derived from the clustering of gland-associated cells.

To quantitatively evaluate segmentation performance, additional testing regions containing a mixture of healthy, malignant, and carcinoma tissue samples were analyzed. The histological regions were manually annotated by expert pathologists and served as the ground-truth reference. Furthermore, a separate training set of 78 labeled tissue samples containing a heterogeneous mix of tissue types was used for parameter optimization of the detection algorithms. Segmentation accuracy was assessed by comparing the automated results with the manual results using the Dice Similarity Coefficient (DSC), both calculated per region and across the entire dataset. These results are summarized in Table 5.

As shown in the table, the average achieved segmentation accuracy was DSC = 0.891 for the automatic segmentation. The DSC value was negatively affected by the presence of challenging cellular structures, while well-preserved healthy regions contributed positively. Difficult cases included densely clustered, dividing, overlapping, or partially sectioned cells, such as those located at the image borders or affected by microtome-induced artifacts (e.g., smearing or tearing). In such instances, only partial cell structures may be visible within a given layer (e.g., faint outlines due to the nucleus of the cell being present in an adjacent section), resulting in morphological features such as the area, perimeter, and pixel intensity falling outside the expected distribution for the respective cell class. These atypical feature values lead to low confidence scores; thus, the detection algorithms tended to discard these ambiguous objects rather than risk false-positive identifications. It is important to note that such cells would also typically be excluded from manual selection for downstream genomic analyses; therefore, this behavior is considered an acceptable trade-off in the scope of our application. Nevertheless, ill-classified objects could still be present in the segmented regions, which have to be accounted for in later stages of the workflow (cell cutting or genomic analysis) or removed by hand once the segmentation is complete.

### 3.4. Manual Selection

**Figure 5:**
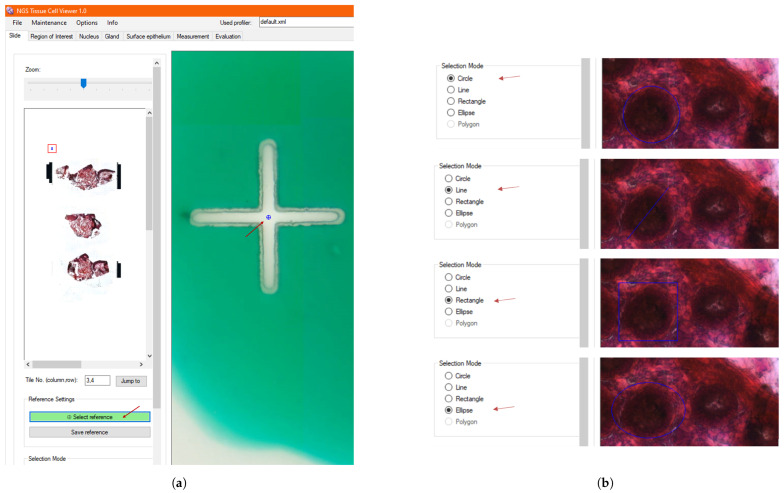
ROI selection process. (**a**) Reference points on the tissue slide are marked and saved to begin the morphological examination. (**b**) Optional shapes (circle, line, square, or ellipse) are selected to define regions of interest (ROIs) to be analyzed further, with the selected shape visible in the image viewer.

**Figure 6:**
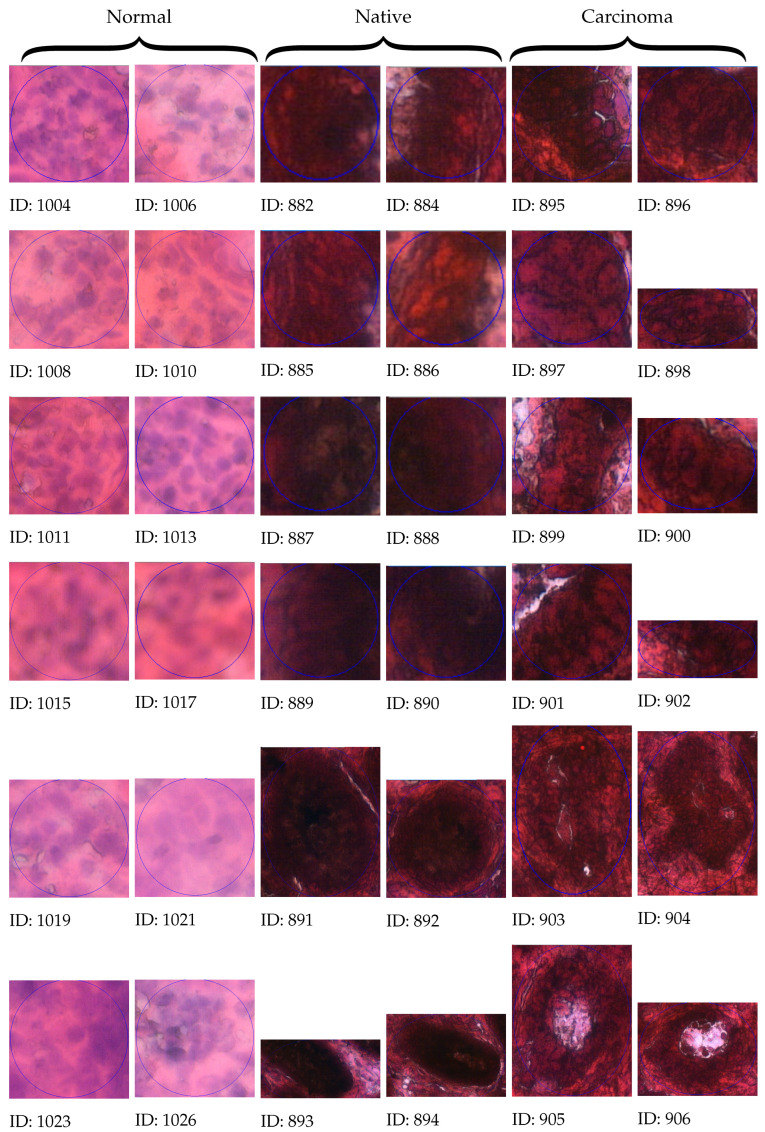
Morphology of the selected regions from normal, native, and carcinoma tissue cells, with 12 samples per tumor state.

Once the reference points are marked on the slide presented in Figure 5a, one can start the morphological examination of the tissue sample and select suspicious areas, as shown in Figure 5b for further genomic analysis—even single-cell areas. Figure 6 displays the morphology of the selected regions from normal, native, and carcinoma tissue samples, with 12-12 samples per tumor state.

### 3.5. Calculated Morphometry

**Figure 7:**
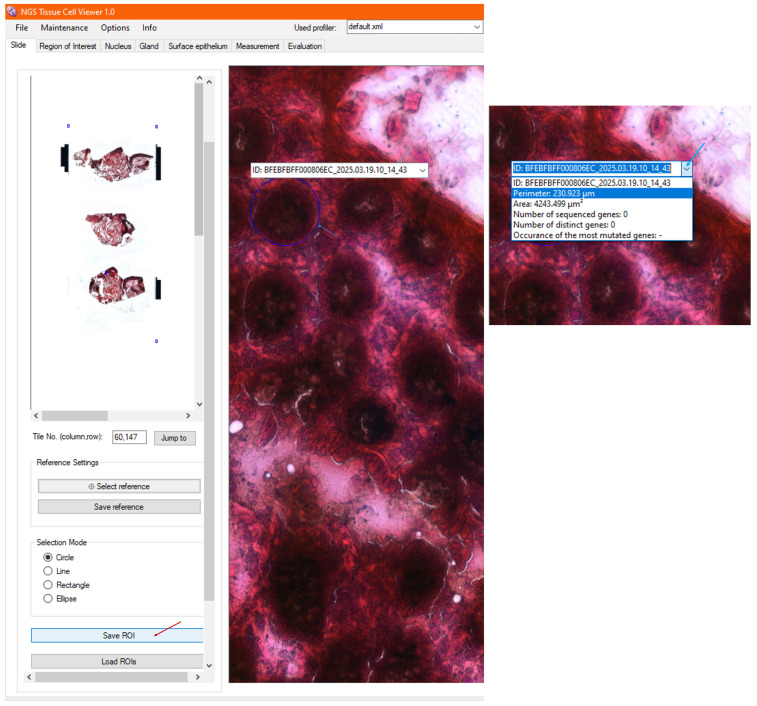
After saving a region of interest (ROI), the image viewer displays the selected ROI, along with calculated morphometric data, such as the perimeter and area, in the annotation list. The saved ROI remains visible across different image resolutions for easy reference and further analysis.

Once the user is satisfied with a selected region, the morphometric parameters of the ROI are also calculated in the background and saved automatically to the fused database when saving an annotation (ROI) in NGS Viewer. Upon exiting a pop-up window and returning to the image viewer shown in Figure 7, the saved ROI selection is fixed, and an additional list window is added above the shape, including the unique identifier of the generated ROI. By scrolling down the list of calculated parameters, one can find morphometric data such as its perimeter and area in micrometers, among other features, which are also displayed in Figure 7.

At this point, while navigating through the image viewer or changing the image resolution, the saved ROI continues to be displayed on the tissue such that one can return to the annotation at any time. To save additional ROIs, the ROI selection (Section 3.4) and saving operations are repeated.

### 3.6. Exporting Single-Cell Morphology and Morphometry to LMD

The parameters of the created ROIs can be saved in an XML file, which serves the purpose of importing those selections into the user interface of the CellCut microdissector. In this way, the selections made on a digital image can be automatically laser-cut from the physical slide using the forward reference points. An exported XML S8 sample file is included in the Supplementary Material. Importing and applying an XML file to the LMD is described in the User Guide of CellCut LMD [32].

### 3.7. Importing Genomic Metadata

The genomic metadata obtained from DNA sequencing of ROI samples can be imported into NGS Viewer for both genomic analysis and visualization. After loading, a confirmation window pops up about the successful import of all the genomic metadata records, line by line. The genomic analysis described in detail in Section 2.8 is automatically executed on the imported genomic metadata.

### 3.8. Loading Archived ROIs

**Figure 8:**
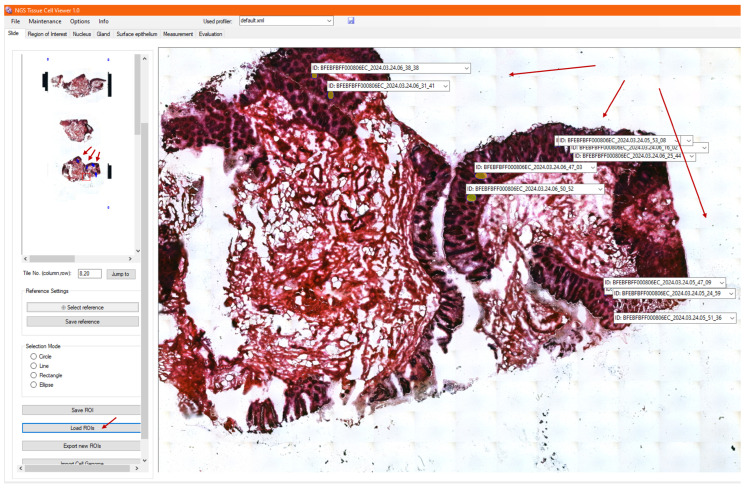
After DNA sequencing, previously saved ROIs can be reloaded in NGS Viewer. The image viewer displays both current and saved annotations, marked with blue frames, to help users efficiently navigate and update the slide without starting from scratch.

After finishing DNA sequencing, it is possible to restore the ROIs that were previously created on the slides in NGS Viewer. This also means that it is not necessary to fully evaluate a given slide at the same time or to start the analysis of the slide from scratch if it has already been started. After importing the ROIs, the program returns to the main screen and jumps to the location of each selected annotation in the image viewer in a row at the current magnification level, as shown in Figure 8.

It also loads the already calculated and analyzed data about the saved ROIs in their annotation lists. In addition, the preview image in the sidebar shows both the current and the loaded annotations marked with blue frames. This way, the user will be aware of where the sample has already been examined and evaluated rather than having to search through the whole image. Once loading is complete, the old annotations can be re-examined, genomic metadata can be added to them, and new ROIs can also be created.

### 3.9. Visualization of Genomic Analysis, Together with Morphology and Morphometry

**Figure 9:**
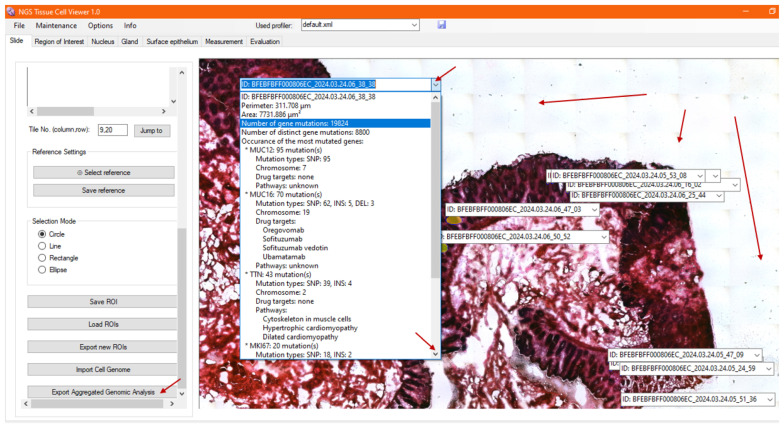
After processing the genomic metadata, NGS Viewer displays the morphology, morphometric measurements, and genomic information for each ROI. This includes mutation details, affected genes, molecular pathways, and drug targets, providing a comprehensive visualization of the tissue sample.

After real-time automatic processing of the imported metadata, the genomic evaluation results can be examined for the high-resolution image at each ROI accordingly. The selected annotations are refreshed; hence, the morphology is now visualized together with its morphometry, as well as its evaluated genomic information in the image viewer, as demonstrated in Figure 9.

Upon opening and scrolling through the annotation list window for the ROI, the genomic information is displayed below the morphometric data, including the total number of mutations detected during DNA sequencing, the number of affected genes, the five most critically mutated genes among the filtered colon-specific genes, their mutation types and occurrences, their known molecular pathways, and the drug targets developed to date, as identified by merging data from international open-source databases. This enables the pathologist to observe the relationships between the mutated genes through their shared molecular pathways—for example, identifying which affected genes participate in the same biological processes, regulatory cycles, or molecular balances. Such correlations support the identification of deeper causal mechanisms and contribute to the establishment of a more precise and robust diagnosis.

Nevertheless, when a digital tissue sample is reopened in the next NGS Viewer usage, the already imported genomic metadata does not need to be imported again; subsequently, the genomic information for the evaluated ROIs will be automatically displayed when the archived ROIs are reloaded.

### 3.10. Visualization of Tumor Foci

**Figure 10:**
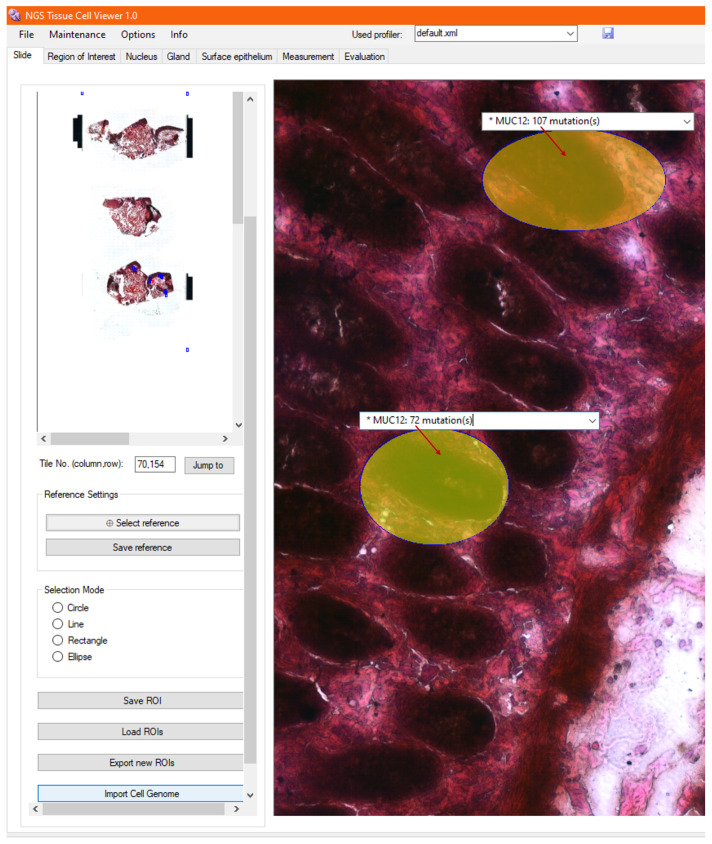
Heatmap overlays on ROI areas based on the severity of detected gene mutations across the sequenced regions. Each ROI is colored according to its aggregated mutation load, with warmer colors (e.g., red/orange) indicating regions with higher mutational intensity and cooler colors (e.g., blue/green) representing regions with lower mutation severity. This visual representation enables comparison of tumor heterogeneity and the identification of tumor foci by highlighting areas with higher mutation intensity, which may be relevant for the delineation of resection margins and understanding localized progression patterns.

To compare the sequenced ROIs (cells) with each other, any list element corresponding to the current examination can be selected from the drop-down annotation lists. When a list item is clicked, the item gets to the top of the list, and the list also closes, as presented in Figure 10. With a closed annotation list and zooming further into the high-resolution image, the heatmap can be viewed in the area of the ROIs, which represent the overall severity of the analyzed gene mutations at a given location.

The heatmap facilitates the comparison of ROI-level heterogeneity within the tumor, supporting the identification of tumor foci by visually progressing along the tumor roughness gradient. Additionally, it assists the pathologist in selecting drugs associated with patient-specific mutations located in the most critically affected, highlighted regions, thereby supporting their consideration during personalized therapy planning. Furthermore, it also supports the oncologist in delineating intervention sites and defining surgical resection margins.

Besides the individual genomic analysis and comparison of single-cell samples, the NGS Viewer enables aggregated analysis of grouped ROIs. The resulting data can be exported in .txt format for further evaluation. The aggregated analysis, as described in detail in Section 2.8, includes the combined or cell-wise genomic evaluation of all sequenced cells associated with a given tissue sample on a chromosome-by-chromosome basis. The analysis determines the total number of gene mutations, the number of affected genes, the proportion of mutated genes relative to the total number of genes per chromosome, and the average number of mutations per gene. Furthermore, for each chromosome, it computes group-level metrics, including the median proportion of mutated genes and the median number of mutations per affected gene. These results can support user-specific research queries and are suitable for visualization through custom-generated plots. A sample of the exportable data format derived from the single-cell samples presented in the above sections is provided in Supplementary Material S9.

### 3.11. NGS Viewer System Testing

During the system’s endurance testing, we observed that the analysis time is primarily influenced by the number of genomic metadata records associated with a given cell. Even the largest sample, containing approximately 56,797 mutation records, was processed in 77 ms, which is within the acceptable delay range of 10–100 ms, demonstrating the system’s classification as a soft real-time system. On average, sequenced cells contain 8422 mutation records, with an average response time of 22.33 ms for their evaluation [39].

In case the system needs to be scaled to handle even larger datasets, based on the identified bottleneck, the system could experience increased memory consumption and/or slower processing times due to the added complexity of managing and analyzing vast amounts of genomic metadata. In particular, the most resource-intensive tasks include data indexing; mutation matching; and database operations such as insert, query, and update, which may lead to delays that exceed the soft real-time constraints. To address these challenges, transitioning from CPU-based to GPU-based genetic analysis, implementing efficient parallel processing, re-optimizing queries, and ensuring robust memory management will be critical in maintaining performance at scale.

## 4. Discussion

Our platform uniquely integrates morphological, morphometrical, and genomic data into a unified analytical space, transforming the way pathologists approach diagnosis and disease understanding. This integrated approach allows for a seamless transition from purely descriptive morphology to a more mechanistic understanding, directly linking specific genetic variations to quantitative phenotypic changes at the cellular and tissue levels. In this discussion, we detail how our platform could be implemented in practice, and we also discuss the potential benefits in clinical applications.

### 4.1. Workflow Integration in Laboratories

The entire workflow, summarized comprehensively in Table 6, seamlessly blends conventional histopathological techniques with high-throughput molecular profiling and advanced robotic handling. Each phase, from the initial biopsy to final therapy planning, is meticulously defined, specifying the necessary specialized equipment—ranging from standard surgical tools to automated slide stainers, laser microdissection systems, and next-generation sequencing (NGS) platforms—with the estimated equipment cost exceeding USD 1.15 million. Key settings such as tissue section thickness, laser parameters, and sequencing coverage depth are highlighted to ensure reproducibility and data quality. Outputs are systematically recorded at each stage, including tissue sections, scanned digital slides, ROI segmentations, extracted cells, raw reads, and annotated gene mutations. The pipeline emphasizes the crucial role of a multidisciplinary team—comprising histologists, immunologists, robotics engineers, bioinformaticians, and oncologists—requiring a staff of at least ten professionals and an estimated processing time of 15 days per sample. This detailed overview aims to provide a transparent and reproducible reference for the implementation and adaptation of similar integrative workflows in precision oncology.

A central tenet of this pipeline is the systematic integration of morphology, morphometry, and genomic data through sequential yet interoperable modules. For instance, high-resolution scanned slides are annotated based on morphological features, which, in turn, guide the precise selection of regions of interest (ROIs) for subsequent laser microdissection and genomic sequencing. This meticulous process ensures a direct and traceable link between cell morphology, its spatial location, and genetic variants, allowing for their correlation through integrated software platforms. The resulting fused data output—encompassing annotated images, morphometric features, and mutation profiles—enables a more holistic understanding of tumor heterogeneity, which is paramount for accurate diagnosis and personalized treatment planning.

The workflow is designed to be compatible with widely used clinical practices embedding cutting-edge digital automation and precision genomic diagnostics. Early stages, such as tissue biopsy, preservation, sectioning, and slide mounting, rely on standard pathology laboratory equipment, requiring minimal infrastructural adaptation. The introduction of robotic slide handling (e.g., UR5 arm) and automated staining (e.g., Gemini stainer) significantly enhances throughput and reproducibility; the initial investment in these systems is justified by potential labor savings and standardized performance. The imaging and data analysis pipeline leverages high-resolution scanning (e.g., Pannoramic P1000 (3DHISTECH, Budapest, Hungary)) and custom image analysis through the NGS Viewer, supporting both segmentation and ROI-based classification with adjustable parameters tailored to specific tissue characteristics. Cell extraction via laser microdissection (LMD) and downstream DNA sequencing (Illumina/Nanopore) enables genomic profiling at single-cell resolution. Critically, the workflow integrates cloud-based or local variant-calling platforms (e.g., BaseSpace or Epi2Me), ensuring interoperability through standardized output formats like .vcf. Subsequent phases of diagnostic reporting and therapy planning are fully integrated in the NGS Viewer environment, allowing for a comprehensive interpretation that fuses visual and molecular data. This framework supports expert-driven interpretation and remains modular enough to accommodate evolving biomarker panels or drug libraries, ensuring reproducibility and personalization for adoption in diverse institutional contexts, including pathology labs with existing molecular diagnostic capabilities. Overall, the workflow offers a high degree of modularity, making partial or full integration feasible, depending on local resources, expertise, and diagnostic focus.

While this platform offers substantial diagnostic potential, its implementation in clinical settings poses notable challenges. The workflow necessitates a series of specialized and high-cost instruments, including slide scanners ($350,000), laser microdissectors (USD 150,000–350,000), and high-throughput sequencers (Illumina: approx. USD 275,000; Nanopore: approx. USD 450,000), in addition to robust server infrastructure for data storage and processing. Furthermore, operating such a sophisticated system demands a multidisciplinary team with specialized expertise in pathology, immunohistochemistry, robotics, sequencing, and bioinformatics. In terms of scalability, the primary bottlenecks are the 3-day tissue preparation phase and the several-hour-long sequencing and variant-calling steps. However, computational data analysis can run in parallel, and the modular nature of the setup enables partial automation and parallelization of tasks. Physically, the system does not strictly require a single integrated laboratory space; equipment components can be distributed across rooms and linked via a local network, provided a contamination-free preparatory environment is ensured for tissue handling. Additionally, strict cooling and climate control are essential, especially for high-load systems like sequencers and servers, which are maintained at 20 °C in the current setup. While the initial setup is resource-intensive, the architecture allows for flexible deployment and gradual integration into existing pathology departments.

### 4.2. Clinical Relevance of the Proposed Framework

Morphological data derived from H&E-stained slides provide foundational phenotypic information, enabling the identification of malignant transformations and other pathological alterations. Complementing this, morphometrical data provide objective, quantifiable measurements of cellular and architectural features, providing a precise assessment of disease progression and statistically significant differences between healthy and diseased cell populations. The fusion of genomic data, such as whole-exome sequencing from specific cellular populations within the slide, introduces a crucial molecular layer. This allows pathologists to identify common mutation patterns, facilitating the recommendation of targeted therapies based on a comprehensive molecular profile. For example, a pathologist can simultaneously view a dysplastic gland, its quantified morphometric parameters (e.g., area and perimeter), and the specific titin (TTN) mutation detected within those very cells.

Although each of these data modalities is present in various forms within current pathology workflows, their integration is often disjoint, leading to inefficiencies and potential inaccuracies.

In current pathological workflows, numerous steps are manual, leading to a lack of automation. Slide digitalization, if performed, is often a time-consuming manual slide-loading and unloading process, especially for larger workloads. Reviewing slides is also a slow process, with results often dependent on the individual pathologist’s expertise, introducing inter-observer variability. Manual morphometry calculations involve repetitive and error-prone steps with a (digital) ruler or a caliper, as each cell must be uniquely labeled and calculated. Furthermore, genetic data, when available, is often disconnected from pathological slides and analyzed using different software. This requires practitioners to cross-reference separate reports manually and switch context frequently, which is both time-consuming and makes the process prone to human error in data transcription or interpretation.

Our integrated platform offers substantial advances in efficiency and accuracy compared to traditional pathologist workflows. By automating the aggregation and direct linkage of morphological, morphometrical, and genomic data, our platform significantly reduces diagnostic response time. Pathologists can rapidly identify key features and receive quantitative support for their observations, which they can immediately correlate with genomic findings from the same regions of interest, eliminating the need to wait for further molecular reports or manually reconcile diverse data sources.

The automated extraction of morphometrical data provides objective, reproducible measurements that would be exceedingly time-consuming or impossible to perform manually. This quantitative layer empowers pathologists with precise metrics that support diagnostic decisions and disease monitoring. By providing quantifiable morphometrical data alongside high-resolution morphology, our system introduces an objective layer that reduces variability in diagnosis and grading across different pathologists, promoting more standardized and reproducible interpretations.

Our unified framework eliminates the need for pathologists to manually reconcile disparate data sources, minimizing the risk of human error in data transcription or interpretation across different modalities. This integrated view enhances the overall accuracy of the diagnosis by providing a comprehensive, cross-referenced understanding of the disease.

The accelerated analysis and standardized workflow lead to more rapid diagnoses and potentially increase the volume of patients who can be effectively managed. Furthermore, this consistent approach promotes uniformity in pathological analysis across different institutions, potentially fostering improved collaboration and inter-institutional diagnostic agreement. This comprehensive and integrated approach represents a significant advancement in diagnostic pathology, facilitating a deeper understanding of disease biology and ultimately enhancing clinical efficiency and patient outcomes.

In addition to aiding pathologists’ work, our approach could also provide additional benefits in precision oncology. While genomics identifies actionable mutations, a fused view can reveal specific morphological or microenvironmental contexts that predict a stronger response to a targeted drug. For example, a TTN-mutated tumor might respond more favorably to treatment if it also exhibits a particular cellular growth pattern or immune cell infiltration. Conversely, the fused data can highlight morphological or architectural features that correlate with resistance to a genome-driven therapy, allowing for an earlier pivot to alternative treatments and sparing patients from ineffective and toxic regimens.

## 5. Conclusions

Our research presents an innovative workflow integration for digital pathology, enabling the aggregation of diverse metadata from independent pathological devices into a unified platform. The NGS Tissue Cell Viewer is a key development, successfully fusing data from tissue sectioning, staining, scanning, segmentation, microdissection, and NGS sequencing, functioning as a tumor-diagnostic decision support system. This integration significantly enhances the efficiency and accuracy of tumor analysis, overcoming the limitations of existing digital pathology solutions that typically analyze this data independently. Unlike traditional methods that treat morphological and genomic data separately, NGS Viewer enables a more holistic and nuanced approach to tumor diagnostics, offering a more seamless diagnostic process and reducing the time and effort required for individual evaluations.

The NGS Viewer empowers simultaneous analysis and visualization of morphological, morphometric, and genomic data, even at the single-cell level. This approach enhances the ability to detect and assess genetic mutations in the context of cellular morphology. It supports precision oncology by enabling patient-specific tumor mutation profiling, displaying essential genomic information, such as the total number of mutations, affected genes, and mutation types, and providing a more comprehensive understanding of the tumor microenvironment. In addition, the identification of associated molecular pathways is also elaborated, which can serve as actionable intervention points. Furthermore, by linking detected gene mutations to known gene-specific drug targets, the platform may assist in assembling personalized treatment strategies. These main functionalities aim to clarify the system’s potential role not only in early cancer detection but also in informing individualized therapeutic decisions and improving clinical outcomes.

Following the first implementation and the integration successes, the current phase of research focuses on scaling up the tissue samples. We have gained server access to colorectal tumor patient samples arriving at the clinic. Additionally, we secured funding for the sequencing of these samples and for the storage of several terabytes of metadata on a dedicated server. As a result, we are currently expanding the sample size and processing it through the established system, scaling up the workflow.

A further limitation is that the evaluation of sequencing metadata involves comparisons with the Hg38 human reference genome, which is, itself, incomplete and continually evolving. We aim to monitor the relevance of these updates, and while this process is currently performed manually, we plan to automate it in the future to ensure that the data remains up-to-date.

In future research, we aim to refine our approach to sequencing data by not only focusing on the quantitative changes in the nucleotides of specific genes but also placing greater emphasis on the locations and qualities of gene mutations. Currently, no complete or standardized collection exists in the literature to clearly associate specific mutations in particular genes with colorectal tumors. Moving forward, we intend to replace this broad approach with an AI-based model trained on large-scale sequencing data, allowing us to search for patterns and provide predictions regarding the severity of mutations based on their locations. Additionally, going down one more genomic level, we plan to identify the type of mutated nucleotides at these filtered locations and provide automated statistical analysis on them. This multi-level approach could significantly improve our understanding of how specific nucleotide mutations contribute to colorectal tumor development and progression, offering deeper insights into the distribution of mutations and their role in the disease.

In addition, using the completed software, we intend to perform automated measurements with an increased sample size, focusing on the native areas to advance our understanding of tumor mechanisms and identify and grasp earlier stages, allowing for easier and more effective strategies to combat cancer and improve patient outcomes.

## Figures and Tables

**Table 1:** Comparative benchmarking of spatial transcriptomics and precision oncology platforms developed by leading companies. The table summarizes the main technologies, core functions, and key technical features of the products offered by 10X Genomics, Bruker Corporation (formerly NanoString Technologies), Oncompass, and Turbine AI.

Vendor	Product	Intended Purpose	Key Features
10X Genomics (Pleasanton, CA, USA)	Chromium Single Cell	Gene expression profiling	Multiomic options in fresh, frozen, and formalin-fixed and paraffin-embedded (FFPE) tissue
10X Genomics	Xenium In Situ platform	Targeting 100s to 1000s of RNAs in individual cells	In fresh, frozen, or FFPE tissue; subcellular profiling; and gene expression
10X Genomics	Visium Spacial	Performing unbiased spatial discovery	Probe-based: HD spatial gene expression
			Poly-A-based: spatial gene expression
10X Genomics	Loupe Browser	Visualization of gene expression	For single-cell transcriptomic data, aligning gene expression spots to histology images, marking, annotating populations, and clustering
Bruker Corporation (Billerica, MA, USA) (NanoString Technologies (Seattle, WA, USA))	nCounter	Tissue object detection, readout creation	Analysis system, panels, and assays
Bruker Corporation (NanoString Technologies)	GeoMx	Highly multiplexed profiling	Digital spatial profiler, panels, and assays
Bruker Corporation (NanoString Technologies)	CosMx	Quantification of up to 1000 RNAs or up to 100 proteins	Smart cyclic in situ hybridization chemistry, single-cell and subcellular resolution, spatial molecular imager, panels, and assays
Bruker Corporation (NanoString Technologies)	AtoMx	Analysis and visualization of spatial multiomics data	Cloud-based spatial informatics platform
Oncompass (Schindellegi, Schwyz, Switzerland)	Precision oncology service	Personalized targeted treatment planning	AI-based determination of both the molecular targets and their associated targeted compounds
Turbine AI (Budapest, Hungary)	Cell model	Drug–cell response simulation	AI-driven predictive modeling for drug discovery and optimization of clinical trial designs

**Table 2:** Empirically optimized parameters for each morphological detection algorithm.

Nucleus Segmentation	Gland Segmentation	Surface Epithelium Segmentation
K-means algorithm	Density-based Hysteresis Snail (DBHS) algorithm	Surface Nucleus Chain-based Algorithm (SNCA)
Gauss filter = true	Use own cell map = true	Minimum sample size = 6000 $\mu m^{2}$
Wallis filter = false	Maximum distance = 18 μm	Maximum sample size = 50,000,000 $\mu m^{2}$
Cell centralization = true	Maximum connection = 14	Full image threshold open mask size = 5 $\mu m^{2}$
Neighborhood = true	Maximum hysteresis distance = 25 μm	Full image threshold open iteration = 5
Mask X size = 5.76 μm	Hysteresis threshold = 80	Maximum hole size = 15,000 $\mu m^{2}$
Mask Y size = 5.76 μm	Maximum pixel count = 10,000	
Zoom level = 2 (Postcalculation)	Get mask = true	
Dilate mask size = 5	Minimum gland area = 1900 $\mu m^{2}$	
Dilate iteration = 1	Maximum gland area = 16,000 $\mu m^{2}$	
Minimum nucleus pixel count = 250	Check compactness = true	
Close iteration 3	Compactness factor = 1.71	
Full slide zoom level = 6	Brightness average delta = 15	
Global threshold = 240	Use rectangular density mask = true	
Surface dilate mask size = 21	Minimum density = 15	
Surface dilate iteration = 1	Density mask size = 20 μm	
	Density map minimum gland area = 5 $\mu m^{2}$	
	Density map maximum gland area = 10,000 $\mu m^{2}$	
	Close mask size = 3 μm	
	Close iteration = 0	

**Table 3:** Aggregated analysis of single-cell genomic data at the chromosome level, showing the number of gene mutations and affected genes per chromosome, along with the proportion of mutated genes and mutation frequency. Data is further aggregated at the tumor stage level (normal, native, and carcinoma) to assess damage across chromosomes, with adjustments for chromosome size variability and tumor stage classification.

Tumor Class: NORM/NAT/CRC		ROI ${\mathrm{ID}}_{1}$				ROI ${\mathrm{ID}}_{2},\ldots ,{\mathrm{ID}}_{n}$	All Cells in Given Tumor Class	
Chrom. Name	Approximate Gene Number of Chromosome	Total Gene Mutations	Affected Distinct Genes	Proportion of Mutated Genes [%]	Approximate Mean of Mutations/Gene	Measured Data of Cells	Median of Mutated Gene Proportions	Median of Mutations per Affected Gene
1 ·2	3000 ·2	$m_{1}^{1}$	$g_{1}^{1}$	$p_{1}^{1}=\left(g_{1}^{1}\;\cdot \;2\right)/\left(3000\;\cdot \;2\right)$	$d_{1}^{1}=m_{1}^{1}/\left(g_{1}^{1}\;\cdot \;2\right)$	…	median ($p_{1}^{1},\ldots ,p_{1}^{n}$)	median ($d_{1}^{1},\ldots ,d_{1}^{n}$)
2 ·2	2500 ·2	$m_{2}^{1}$	$g_{2}^{1}$	$p_{2}^{1}=\left(g_{2}^{1}\;\cdot \;2\right)/\left(2500\;\cdot \;2\right)$	$d_{2}^{1}=m_{2}^{1}/\left(g_{2}^{1}\;\cdot \;2\right)$	…	median ($p_{2}^{1},\ldots ,p_{2}^{n}$)	median ($d_{2}^{1},\ldots ,d_{2}^{n}$)
3 ·2	1900 ·2	$m_{3}^{1}$	$g_{3}^{1}$	$p_{3}^{1}=\left(g_{3}^{1}\;\cdot \;2\right)/\left(1900\;\cdot \;2\right)$	$d_{3}^{1}=m_{3}^{1}/\left(g_{3}^{1}\;\cdot \;2\right)$	…	median ($p_{3}^{1},\ldots ,p_{3}^{n}$)	median ($d_{3}^{1},\ldots ,d_{3}^{n}$)
further chromosomes…						…	…	…
21 ·2	400 ·2	$m_{21}^{1}$	$g_{21}^{1}$	$p_{21}^{1}=\left(g_{21}^{1}\;\cdot \;2\right)/\left(400\;\cdot \;2\right)$	$d_{21}^{1}=m_{21}^{1}/\left(g_{21}^{1}\;\cdot \;2\right)$	…	median ($p_{21}^{1},\ldots ,p_{21}^{n}$)	median ($d_{21}^{1},\ldots ,d_{21}^{n}$)
X ·1	1400 ·1	$m_{x}^{1}$	$g_{x}^{1}$	$p_{x}^{1}=\left(g_{x}^{1}\;\cdot \;1\right)/\left(1400\;\cdot \;1\right)$	$d_{x}^{1}=m_{x}^{1}/\left(g_{x}^{1}\;\cdot \;1\right)$	…	median ($p_{x}^{1},\ldots ,p_{x}^{n}$)	median ($d_{x}^{1}\ldots ,d_{x}^{n}$)
Y ·1	200 ·1	$m_{y}^{1}$	$g_{y}^{1}$	$p_{y}^{1}=\left(g_{y}^{1}\;\cdot \;1\right)/\left(200\;\cdot \;1\right)$	$d_{y}^{1}=m_{y}^{1}/\left(g_{y}^{1}\;\cdot \;1\right)$	…	median ($p_{y}^{1},\ldots ,p_{y}^{n}$)	median ($d_{y}^{1},\ldots ,d_{y}^{n}$)

**Table 4:** Minimal system requirements to use NGS Tissue Cell Viewer.

PC Component	Details
Operating system	Microsoft Windows 10 (64-bit recommended)
Execution environment	Microsoft .Net Framework, ≥version 4.8.04084
Disk space	300 MB free space for program and 10 GB for local slide storage (1 digital slide size is 0.5–8 GB, depending on resolution)
RAM	≥2 GB, 1.6 GHz (4 GB recommended)
CPU	≥Intel(R) Core(TM) i5 or equivalent AMD processor
Graphics card memory	512 MB (1 GB recommended)
Screen resolution	19″, 1280 × 1024 resolution, 141 DPI

**Table 5:** Performance of the automatic segmentation across a heterogeneous set of tissue samples. For each sample, the table lists the number of cells detected by the algorithm, the number of manually annotated cells (control), and the number of cells that were missed (false negatives) after confidence-based filtering. The Dice similarity coefficient (DSC) is reported per sample as a quantitative measure of segmentation accuracy. The average DSC across all samples was 0.891.

Tissue Sample	Number of Detected Cells	Manual Number of Cell Annotations	Number of Filtered, Missed Cells (False Negative)	DSC
Sample A	160	207	47	0.872
Sample B	261	269	8	0.985
Sample C	176	197	21	0.944
Sample D	367	444	77	0.905
Sample E	545	706	161	0.871
Sample F	716	872	156	0.902
Sample G	285	410	125	0.820
Sample H	431	484	53	0.890
Sample I	256	288	32	0.889
Sample J	89	117	28	0.864
Total	3286	3994	708	0.891

**Table 6:** Integration details across the cyber-physical tumor diagnostic workflow.

Workflow Phase	Specialized Equipment, Chemistry	Approx. Mean Price [USD] (in 2025)	Important Settings	Output	Technical Expertise	Time Requirement
Biopsy	Clinical setup, scalpel	N/A	Standard biopsy protocol, resection margins including native region	Raw tissue sample	Surgeon, nurse, staff	1–2 h
Tissue preservation	Ultra-low-temperature (ULT) freezer, cryogenic tube/formalin, paraffin	∼15,000/∼850	Snap-frozen tissue stored at −80 °C, while paraffin block stored at −4 °C in a freezer.	Snap-frozen/FFPE tissue block	Lab technician	3 days
Tissue sectioning	Automatic microtome	∼30,000	Preventing tissue thawing during handling. Section thickness proportionality to examining cell type (Snap-frozen: 10–20 µm, FFPE: 4–6 µm) and uniformity of sections.	Tissue sections	Histologist supervision	0.5 h
Section mounting	Warm water bath for surface stretching of tissue sections, slide-drying hot plate	∼1000 + 1600	Membrane slides (MMI) used instead of classic glass slides for LMD compatibility. To ensure that no air bubbles are trapped, a brush is used to flatten.	Sections fixed on slides	Lab technician	1.5 h
Slide transferring	UR5 robotic arm (Universal Robots USA, Novi, MI, USA) with custom magazine and slide manipulator	∼50,000	Accurate, damage-free membrane slide placement into downstream modules.	Loaded slides	Robotics engineer supervision	10 min
Slide staining	Gemini automated stainer (Thermo Fisher Scientific, Waltham, MA, USA), H&E stainer kit/IHC panel kit	∼60,000 + ∼150–5000	Staining and IHC protocols	Contrast-enhanced, stained sections	Immunologist supervision	3 h (protocol-dependent)
Slide scanning	Pannoramic P1000 scanner (3DHistech, Budapest, Hungary)	∼350,000	Use of obtained custom settings profile for membrane slides.	High-resolution digital slide images (.MRXS)	Lab technician supervision	5–30 min (resolution-dependent)
Image analysis: segmentation and classification	NGS Viewer	N/A	Fine-tuning of algorithm parameters for examined tissue type.	Selected ROIs (e.g., cells), their morphology, calculated morphometry saved in DB and exportable ROI parameters for excision (.xml)	Bioinformatician supervision	10–20 min
Cell extraction (step required only for single-cell-level analysis)	CellCut LMD (MMI)	∼150,000–200,000	Laser velocity, focus, and power adjustment based on tissue type and section thickness. 2x cutting repeat.	Isolated cells in Eppendorf capsules	Lab technician	0.5 h
DNA sequencing: electrical signal reading and base calling	NextSeq 500 (Illumina, Inc., San Diego, CA, USA)+kit for exome of single cells/PromethION (Nanopore, Oxford, UK)+kit for whole genome of bulk + server	∼275,000+3000/run/ ∼450,000+1000/run + 10000	≥40 TB data storage. Quality checking for sequencing depth and uniformity of coverage.	Raw signal reads (.POD5) and nucleotide sequences (.FASTQ)	Lab technician	5–10 days
Variant calling: contig alignment and variant calling	BaseSpace (Illumina)/Epi2Me (Nanopore)	Usage-based (0.035/GB)/free	≥20 TB data storage. Flowcell + chemistry compatible model use. Comparison with the latest human reference genome.	Exportable genomic metadata of ClinVar DB annotated gene mutations (.vcf)	Bioinformatician supervision	0.5 day
Diagnosis reporting	NGS Viewer	N/A	Fused genomic metadata analysis and visualization by reopening saved ROI morphology and morphometry data and importing .vcf variants. Correlation examination via the listed molecular pathways of the mutated genes.	Support of diagnostic conclusion establishment	Pathologists	30–40 min
Therapy planning	NGS Viewer	N/A	Selection of listed gene-specific drugs that target the most critically mutated genes.	Personalized treatment plan support	Oncologist board and attending physician	20–30 min
Total		≥1.15M			Staff of ≥10 people	∼10–15 days

## Data Availability

Due to privacy issues, we have not made the full dataset available, including the digital images of medical tissue sections and the single-cell sequencing part of this publication. The data were used according to the written consent provided by participants, without compromising their anonymity. Upon request, we can provide access to the data with permission for peer review.

## Supplementary Materials

The following supporting information can be downloaded at: https://www.mdpi.com/article/10.3390/s25144465/s1, S1: Written informed consent template signed by patients involved in the study; S2: Importable scanning protocol containing scanner parameter settings; S3: Detailed usage steps of NGS Viewer; S4: Workflow feasibility proof that has already been patented; S5: Digitized tissue sample from normal tissue area; S6: Digitized tissue sample from native tissue area; S7: Digitized tissue sample from cancerous tissue area; S8: Exported annotation from NGS Viewer that can be imported into the user interface of MMI’s LMD for automatic laser cutting of the annotations; S9: Gene mutations of ROIs analyzed per chromosome in the NORM, NAT, and CRC samples; S10: Disclosure of potential conflicts of interest signed by the authors.

## Abbreviations

The following abbreviations are used in this manuscript:

A	Adenine
API	Application programming interface
C	Cytosine
CRC	Carcinoma (cancerous tissue)
DB	Database
DEL	Deletion of nucleotide sequence
DNA	Deoxyribonucleic acid
DSC	Dice similarity coefficient
FFPE	Formalin-fixed and paraffin-embedded
G	Guanine
GUI	Graphical user interface
HG38	Human reference genome 38
IHC	Immunohistochemical
INS	Insertion of nucleotide sequence
LMD	Laser microdissection machine
MMI	Molecular Machines & Industries Ltd.
NAT	Native tissue (the nearest intact tissue sample to cancer)
NGS	Next-generation sequencing
NORM	Normal tissue (healthy)
ROI	Region of interest
SNP	Single-nucleotide polymorphism
T	Thymine
TME	Tumor microenvironment
TTN	Titin protein
ULT	Ultra-low temperature
U	Uracil

## References

[1] Fischer E. (2020). Nuclear Morphology and the Biology of Cancer Cells. Acta Cytol..

[2] Folkman J. (1971). Tumor Angiogenesis: Therapeutic Implications. N. Engl. J. Med..

[3] Kinzler K., Vogelstein B. (1996). Lessons from Hereditary Colorectal Cancer. Cell.

[4] Garber M.E., Troyanskaya O.G., Schluens K., Petersen S., Thaesler Z., Pacyna-Gengelbach M., van de Rijn M., Rosen G.D., Perou C.M., Whyte R.I. (2001). Diversity of gene expression in adenocarcinoma of the lung. Proc. Natl. Acad. Sci. USA.

[5] Perou C., Sørlie T., Eisen M., van de Rijn M., Jeffrey S., Rees C., Pollack J., Ross D., Johnsen H., Aksle N.L. (2000). Molecular portraits of human breast tumours. Nature.

[6] Yachida S., Jones S., Bozic I., Antal T., Leary R., Fu B., Kamiyama M., Hruban R., Eshleman J., Nowak M. (2010). Distant Metastasis Occurs Late during the Genetic Evolution of Pancreatic Cancer. Nature.

[7] Jones S., Zhang X., Parsons D., Lin J., Leary R., Angenendt P., Mankoo P., Carter H., Kamiyama H., Jimeno A. (2008). Core Signaling Pathways in Human Pancreatic Cancers Revealed by Global Genomic Analyses. Science.

[8] Burrell R.A., McGranahan N., Bartek J., Swanton C. (2013). The Causes and Consequences of Genetic Heterogeneity in Cancer Evolution. Nature.

[9] Gerlinger M., Horswell S., Larkin J., Rowan A., Salm M., Varela I., Fisher R., McGranahan N., Matthews N., Santos C. (2014). Genomic Architecture and Evolution of Clear Cell Renal Cell Carcinomas Defined by Multiregion Sequencing. Nat. Genet..

[10] Knoche S.M., Larson A.C., Sliker B.H., Poelaert B.J., Solheim J.C. (2021). The role of tumor heterogeneity in immune-tumor interactions. Cancer Metastasis Rev..

[11] Germanà E., Ludovica P., Pizzimenti C., Ballato M., Pierconti F., Tuccari G., Ieni A., Giuffrè G., Fadda G., Fiorentino V. (2024). Programmed Cell Death Ligand 1 (PD-L1) Immunohistochemical Expression in Advanced Urothelial Bladder Carcinoma: An Updated Review with Clinical and Pathological Implications. Int. J. Mol. Sci..

[12] Beca F., Polyak K. (2016). Intratumor Heterogeneity in Breast Cancer. Novel Biomarkers in the Continuum of Breast Cancer.

[13] 10X Genomics Compare Products. https://www.10xgenomics.com/products.

[14] 10X Genomics In Situ Gene Experssion. https://www.10xgenomics.com/support/in-situ-gene-expression.

[15] 10X Genomics Find Product User Guide for In Situ Gene Expression. https://www.10xgenomics.com/support/user-guides/in-situ-gene-expression?menu%5bproductNames%5d=In%20Situ%20Gene%20Expression.

[16] NanoString, A Bruker Company What is Spatial Biology?. 2022..

[17] NanoString, A Bruker Company What is Spatial Transcriptomics?. https://nanostring.com/research-focus/spatial-transcriptomics/.

[18] Oncompass Gmbh Precision Oncology Program. https://oncompassmedicine.com/about-the-process#.

[19] Papp O., Jordán V., Hetey S., Balázs R., Kaszás V., Bartha Á., Ordasi N.N., Kamp S., Farkas B., Mettetal J. (2024). Network-driven cancer cell avatars for combination discovery and biomarker identification for DNA damage response inhibitors. NPJ Syst. Biol. Appl..

[20] Mareček-Kolibiský M., Janík S., Mĺkva M., Szabó P., Czifra G. (2024). Human-Machine Co-Working for Socially Sustainable Manufacturing in Industry 4.0. Acta Polytech. Hung..

[21] Kozlovszky M., Hegedűs K., Szenasi S., Kiszler G., Wichmann B., Bándi I., Eigner G., Sas P., Kovács L., Garaguly Z. Parameter assisted HE colored tissue image classification. Proceedings of the 2013 IEEE 17th International Conference on Intelligent Engineering Systems (INES).

[22] Thermo Scientific Thermo Scientific Gemini AS Operator Guide Issue 10. https://www.medwrench.com/documents/view/15154/thermo-scientific-gemini-as-operator-guide-issue-10.

[23] Cardiff R.D., Miller C.H., Munn R.J. (2014). Manual Hematoxylin and Eosin Staining of Mouse Tissue Sections. Cold Spring Harb. Protoc..

[24] Epredia ClearVue Coverslipper. https://www.epredia.com/products/histology-instruments/staining-and-coverslipping/clearvue.

[25] Universal Robots A/S UR5e. https://www.universal-robots.com/products/ur5e/.

[26] Kucarov M.D., Molnár B., Kozlovszky M. Robot Instead of Laboratory Technicians - Slide Holder Detection and 3D Position Determination by Robotic Arm. Proceedings of the 2022 IEEE 26th International Conference on Intelligent Engineering Systems (INES).

[27] Kucarov M.D., Takács M., Molnár B., Kozlovszky M. Transparent Slide Detection and Gripper Design for Slide Transport by Robotic Arm. Proceedings of the 2022 IEEE 22nd International Symposium on Computational Intelligence and Informatics and 8th IEEE International Conference on Recent Achievements in Mechatronics, Automation, Computer Science and Robotics (CINTI-MACRo).

[28] Kucarov M.D., Molnár B., Kozlovszky M. Calibration of Robotic Arm for Workstation Installation in Changing Environment—Comparison of Manual, Mechanic, and Automatic Calibration. Proceedings of the 2023 IEEE 17th International Symposium on Applied Computational Intelligence and Informatics (SACI).

[29] 3DHISTECH (2018). Pannoramic 1000 User’s Guide. https://assets.thermofisher.com/TFS-Assets/APD/Product-Guides/US-Only-P1000-BF-Users-Guide-EN-2018-04.pdf.

[30] Kucarov M.D., Molnár B., Kozlovszky M. (2024). Localization and Conversion of Single Cell Positions from Static High-Resolution Digital Images to Lasermicrodissector Coordinate System through Utilization of References and 2D Transformation Techniques. Acta Polytech. Hung..

[31] Kucarov M.D., Molnár B., Kozlovszky M. Single Cell Position Determination and Transformation From Static High-resolution Digital Image To Laser-microdissector Coordinate System Using Image Processing Techniques. Proceedings of the 2023 IEEE 17th International Symposium on Applied Computational Intelligence and Informatics (SACI).

[32] Molecular Machines, Industries GmbH (2020). mmi CellCut User Manual. https://scopem.ethz.ch/center/instruments-alphabetical/nikon-mm1-md.html.

[33] Illumina, Incorporation MiniSeq Sequencing System Applications and Methods. https://www.illumina.com/systems/sequencing-platforms/miniseq/applications.html.

[34] Oxford Nanopore Technologies plc PromethION 24/48. https://nanoporetech.com/products/sequence/promethion-24-48.

[35] Illumina, Incorporation Illumina DRAGEN Bio-IT Platform v3.6 User Guide. https://support.illumina.com/content/dam/illumina-support/documents/documentation/software_documentation/dragen-bio-it/dragen-bio-it-platform-v3.6-user-guide-1000000128306-00.pdf.

[36] Illumina, Incorporation Germline Variant Small Hard Filtering. https://jp.support.illumina.com/content/dam/illumina-support/help/Illumina_DRAGEN_Bio_IT_Platform_v3_7_1000000141465/Content/SW/Informatics/Dragen/GPipelineVarCalFilt_fDG.htm.

[37] Kucarov M.D., Molnár B., Kozlovszky M. Patho-Genomics Fusioned Database Schema and Optimisation for Automatic Pathology Workflow. Proceedings of the 2023 IEEE 21st World Symposium on Applied Machine Intelligence and Informatics (SAMI).

[38] Kanehisa Laboratories KEGG: Kyoto Encyclopedia of Genes and Genomes. https://www.genome.jp/kegg/.

[39] Kucarov M.D., Molnár B., Kozlovszky M. Integration of NGS Genomic Metadata Analysis with Open-source Genomic Databases for Single Cell Tissue Samples. Proceedings of the 2025 IEEE 12th International Conference on Computational Cybernetics and Cyber-Medical Systems (ICCC).

[40] DocCheck Community GmbH (2024). Chromosom. https://flexikon.doccheck.com/de/Chromosom.

[41] National Center for Biotechnology Information (NCBI) (2016). Chromosome Map. https://www.ncbi.nlm.nih.gov/books/NBK22266/.

[42] The Npgsql Development Team Npgsql Getting Started. https://www.npgsql.org/doc/index.html.

