# Supplementary material for: Enhanced Tumor Diagnostics via Cyber-Physical Workflow: Integrating Morphology, Morphometry, and Genomic MultimodalData Analysis and Visualization in Digital Pathology
Source: Sensors (Basel). 2025 Jul 17;25(14):4465. doi: 10.3390/s25144465 (PMC12300765; doi:10.3390/s25144465)
Supplement: Supplementary file 1 [file sensors-25-04465-s001.zip › S1_Written_Informed_Consent__translated.pdf]

WRITTEN INFORMED CONSENT

Title: Hungarian Oncogenome and personalized tumor diagnostics and therapy national programme (NVKP_16-1-2016-0004)

Professional leader: Prof. Dr. András Matolcsy & Dr. Zoltán Máthé
Place of the study: Semmelweis University, Faculty of Medicine
Department of Pathology and Experimental Research
Department of Surgery and Transplantation

I declare that I have read the enclosed patient information sheet and I consent to the use of the sample taken from me for research purposes during the implementation of the research project NVKP_16-1-2016-0004. With my signature, I certify that the details of the examination have been explained, any questions I may have have been answered, and that I voluntarily consent to the examination. I am aware that the study was reviewed and approved by an independent Ethics Committee. I agree that the data related to my illness will be used for the purpose of scientific analysis - keeping my personal data confidential.

I am aware that I can withdraw my consent to the study at any time without a specified reason, in verbal or in written way.

Name of the patient (capital letters): ……………………………………………………………

Mother's name: ……………………………………………………………

Place of birth: ……………………………………………………………

Date of birth: ……………………………………………………………

Social Security code: ……………………………………………………………

Address: ……………………………………………………………

Patient's signature: ……………………………………………………………

Date: ……………………………………………………………

If case the patient is incapacitated or has limited capacity to act, to the patient's legal representative

Name (capital letters): ……………………………………………………………

Signature: ……………………………………………………………

Date: ……………………………………………………………

Informing doctor (capital letters)	………………………………………………………………
Signature:	………………………………………………………………
Date:	………………………………………………………………
