# Supplementary material for: Enhanced Tumor Diagnostics via Cyber-Physical Workflow: Integrating Morphology, Morphometry, and Genomic MultimodalData Analysis and Visualization in Digital Pathology
Source: Sensors (Basel). 2025 Jul 17;25(14):4465. doi: 10.3390/s25144465 (PMC12300765; doi:10.3390/s25144465)
Supplement: Supplementary file 1 [file sensors-25-04465-s001.zip › S3_NGS_Viewer_user_interactions.pdf]

S3 - Operational Steps in NGS Viewer: A Brief Technical Guide for User Interactions

June 25, 2025

In this supplementary material, we add a more detailed technical description of the use of the NGS viewer application.

0.1 Opening digital membrane slides

Launching the NGS Viewer application takes one to the main screen. In the menu bar of the main screen, choosing the File menu and clicking on the "Open slide", a library browser window will pop up, which can be used to navigate to the folder containing the MRXS-type digital slides to work with. After selecting the location of any of the slides on the computer, NGS Viewer will load both a thumbnail preview of the entire section into the sidebar (left side) and a high-resolution subpart of the digital slide into the image viewer (right side of the main screen). Once a slide is loaded, several processing options become active.

0.2 Selecting tissue morphology

In the Viewer, one can click anywhere on the preview images above to navigate to the desired part of the tissue that they wish to examine. A red area selection frame will then jump to that region, and the image viewer on the right side of the main screen will display the selected area at the current magnification level. The magnification level (zoom level) can be changed using the Zoom slider in the sidebar. Dragging the slider to the right increases the resolution, while dragging it to the left decreases the resolution. By default, the slider displays the selected tissue area at medium magnification. One can also zoom in and out by clicking in the image viewer and then using the mouse scroll wheel.

0.2.1 Selecting ROI

Once the reference points are marked on the slide, selecting the ROIs can be performed. To do so, the following steps need to be taken:

1. Locating the area to be selected on the image viewer at any resolution.

2. Selecting the annotation shape from the "Selection mode" options in the sidebar. One can select a circle, line, square, or ellipse, as these shapes can be laser cut out later.

3. To start the selection, press and hold the left mouse button while moving the mouse diagonally to define the size of the shape.

4. Once the size of the shape is sufficient, release the left mouse button. This completes the selection.

5. If the user wants to select another area, steps 3 to 4 have to be repeated. If the selection is correct, one can proceed with saving the ROI.

0.3 Calculating morphometry

Once the selected region is saved, the morphometric parameters of the region are also calculated in the background and saved automatically to the fused database. A pop-up window will appear with the resulting ROI image. This window contains the following information about the area being analyzed:

- Selection width in pixels.
- Selection height in pixels.
- The RGB color space values of the pixel under the mouse pointer, separately:
  - Red color intensity value,
  - Green color intensity value,
  - Blue color intensity value.
- Position of the mouse pointer.

Upon exiting a pop-up window and returning to the image viewer, the saved ROI selection has been fixed, and an additional list window has been added above the shape. By scrolling down the list of calculated parameters, one can find morphometric data such as its perimeter and area in micrometers, among other features.

0.4 Exporting single cell morphology, morphometry to microdissection

To execute this, the following steps describe the process:

1. Clicking on the "Export new ROIs" button on the sidebar

2. In the pop-up window, the location where one wants to save the file should be selected.

3. Changing the XML file name if required.

4. Clicking on the Save button to create the file.

5. After saving, a confirmation window will pop up to confirm the successful creation of the XML file that one can acknowledge by clicking on proceed.

One will find the file in the selected save location.

0.5 Importing genomic metadata

To import genomic metadata files, the following steps must be performed:

1. Clicking on the "Import Genome" button on the sidebar.

2. In the pop-up window, one can search for the genomic txt file(s) with a unique identifier of the sequenced ROI to be loaded.

3. By clicking on the "Open" button in the pop-up window, the content of the selected file is imported into the database of NGS Viewer.

0.6 Loading archived ROIs

It can be obtained by opening the same slide examined previously and clicking on the "Load ROIs" button on the sidebar, which pops up a loading window. The "annotations" field of the window lists the unique identifiers of the archived ROIs queried from the database, line by line. A user can select one, multiple, or even all the annotations at once by ticking the "Select all" option. Once one has selected the annotation(s) to load, click on the Load button to import them.

0.7 Visualizing genomic analysis together with morphology and morphometry

A "Load ROI" window pops up right after the genomic evaluation has finished. Ticking the checkbox beside the unique ID of the evaluated ROI updates the ROI visualization.
