# Supplementary material for: Enhanced Tumor Diagnostics via Cyber-Physical Workflow: Integrating Morphology, Morphometry, and Genomic MultimodalData Analysis and Visualization in Digital Pathology
Source: Sensors (Basel). 2025 Jul 17;25(14):4465. doi: 10.3390/s25144465 (PMC12300765; doi:10.3390/s25144465)
Supplement: Supplementary file 1 [file sensors-25-04465-s001.zip › S9_Gene_mutations_of_cells_per_chrs.pdf]

Table S9. Gene mutations of the sequenced cells evaluated per chromosome in the normal (green), native (yellow) and carcinoma (red) samples. Normal contains all detected, healthy mutations and serves as a reference. While native and carcinoma are already filtered with the reference, they contain only the somatic (tumor-related) gene mutations.

NORMAL	ROI ID	1003				1005				1007			
Chromosome	Approximate gene number of chromosome	Total gene mutations	Affected distinct genes	Proportion of mutated genes	Approximate mean of mutations per gene	Total gene mutations	Affected distinct genes	Proportion of mutated genes	Approximate mean of mutations per gene	Total gene mutations	Affected distinct genes	Proportion of mutated genes	Approximate mean of mutations per gene
1	3000	222	143	4.77%	0.78	1624	744	24.80%	1.09	99	64	2.13%	0.77
2	2500	167	108	4.32%	0.77	851	400	16.00%	1.06	45	36	1.44%	0.63
3	1900	137	100	5.26%	0.69	617	350	18.42%	0.88	23	20	1.05%	0.58
4	1600	97	67	4.19%	0.72	322	196	12.25%	0.82	27	24	1.50%	0.56
5	1700	87	61	3.59%	0.71	572	296	17.41%	0.97	24	23	1.35%	0.52
6	1900	183	114	6.00%	0.80	1114	425	22.37%	1.31	31	22	1.16%	0.70
7	1800	113	75	4.17%	0.75	414	238	13.22%	0.87	48	32	1.78%	0.75
8	1400	65	47	3.36%	0.69	323	178	12.71%	0.91	31	25	1.79%	0.62
9	1400	126	84	6.00%	0.75	479	238	17.00%	1.01	42	28	2.00%	0.75
10	1400	101	64	4.57%	0.79	339	205	14.64%	0.83	34	28	2.00%	0.61
11	2000	192	127	6.35%	0.76	835	404	20.20%	1.03	80	52	2.60%	0.77
12	1600	132	97	6.06%	0.68	523	303	18.94%	0.86	41	37	2.31%	0.55
13	800	41	29	3.63%	0.71	180	103	12.88%	0.87	12	10	1.25%	0.60
14	1200	65	42	3.50%	0.77	326	186	15.50%	0.88	16	16	1.33%	0.50
15	1200	121	73	6.08%	0.83	342	169	14.08%	1.01	27	19	1.58%	0.71
16	1300	76	61	4.69%	0.62	507	263	20.23%	0.96	23	17	1.31%	0.68
17	1600	152	99	6.19%	0.77	507	295	18.44%	0.86	26	23	1.44%	0.57
18	600	43	23	3.83%	0.93	247	115	19.17%	1.07	19	15	2.50%	0.63
19	1700	226	144	8.47%	0.78	917	411	24.18%	1.12	97	51	3.00%	0.95
20	900	55	44	4.89%	0.63	236	163	18.11%	0.72	17	16	1.78%	0.53
21	400	27	21	5.25%	0.64	127	60	15.00%	1.06	1	1	0.25%	0.50
22	800	77	49	6.13%	0.79	259	137	17.13%	0.95	9	6	0.75%	0.75
X	1400	68	50	3.57%	0.68	326	204	14.57%	0.80	26	24	1.71%	0.54
Y	200	0	0	0.00%	0.00	0	0	0.00%	0.00	0	0	0.00%	0.00

…

Chromosome	1021				1023				1025				all normal cells	
	Total gene mutations	Affected distinct genes	Proportion of mutated genes	Approximate mean of mutations per gene	Total gene mutations	Affected distinct genes	Proportion of mutated genes	Approximate mean of mutations per gene	Total gene mutations	Affected distinct genes	Proportion of mutated genes	Approximate mean of mutations per gene	Median of mutated genes proportions	Median of mutations per affected gene approximation
1	5123	1218	40.60%	2.10	608	388	12.93%	0.78	87	53	1.77%	0.82	**6.38%**	**0.81**
2	3950	976	39.04%	2.02	511	278	11.12%	0.92	77	42	1.68%	0.92	**5.64%**	**0.81**
3	4328	870	45.79%	2.49	356	224	11.79%	0.79	53	37	1.95%	0.72	**7.55%**	**0.80**
4	3230	733	45.81%	2.20	298	167	10.44%	0.89	17	12	0.75%	0.71	**4.09%**	**0.78**
5	2635	685	40.29%	1.92	273	177	10.41%	0.77	71	43	2.53%	0.83	**4.21%**	**0.77**
6	2505	669	35.21%	1.87	413	224	11.79%	0.92	35	23	1.21%	0.76	**5.71%**	**0.92**
7	4109	884	49.11%	2.32	287	163	9.06%	0.88	51	24	1.33%	1.06	**5.17%**	**0.88**
8	2388	530	37.86%	2.25	223	133	9.50%	0.84	36	24	1.71%	0.75	**3.43%**	**0.81**
9	1634	431	30.79%	1.90	219	142	10.14%	0.77	36	18	1.29%	1.00	**6.00%**	**0.81**
10	2800	612	43.71%	2.29	255	171	12.21%	0.75	39	22	1.57%	0.89	**5.71%**	**0.82**
11	4567	992	49.60%	2.30	423	251	12.55%	0.84	55	30	1.50%	0.92	**6.33%**	**0.85**
12	2727	678	42.38%	2.01	344	203	12.69%	0.85	42	33	2.06%	0.64	**6.84%**	**0.75**
13	2003	396	49.50%	2.53	121	66	8.25%	0.92	10	10	1.25%	0.50	**5.13%**	**0.84**
14	2006	478	39.83%	2.10	205	125	10.42%	0.82	32	22	1.83%	0.73	**5.42%**	**0.79**
15	2119	485	40.42%	2.18	184	104	8.67%	0.88	33	24	2.00%	0.69	**5.96%**	**0.81**
16	2034	471	36.23%	2.16	176	120	9.23%	0.73	65	45	3.46%	0.72	**5.69%**	**0.72**
17	2619	654	40.88%	2.00	285	183	11.44%	0.78	121	62	3.88%	0.98	**7.41%**	**0.82**
18	812	222	37.00%	1.83	136	73	12.17%	0.93	8	6	1.00%	0.67	**6.42%**	**0.88**
19	1626	485	28.53%	1.68	450	229	13.47%	0.98	56	25	1.47%	1.12	**8.15%**	**1.03**
20	964	312	34.67%	1.54	109	79	8.78%	0.69	22	14	1.56%	0.79	**5.33%**	**0.76**
21	542	152	38.00%	1.78	62	41	10.25%	0.76	9	3	0.75%	1.50	**6.00%**	**0.81**
22	1114	305	38.13%	1.83	96	55	6.88%	0.87	9	4	0.50%	1.13	**6.44%**	**0.83**
X	952	372	26.57%	1.28	184	130	9.29%	0.71	11	11	0.79%	0.50	**3.07%**	**0.66**
Y	10	5	2.50%	1.00	11	1	0.50%	5.50	0	0	0.00%	0.00	**0.00%**	**0.00**

NATIVE	ROI ID	882				885				887			
Chromosome	Approximate gene number of chromosome	Total gene mutations	Affected distinct genes	Proportion of mutated genes	Approximate mean of mutations per gene	Total gene mutations	Affected distinct genes	Proportion of mutated genes	Approximate mean of mutations per gene	Total gene mutations	Affected distinct genes	Proportion of mutated genes	Approximate mean of mutations per gene
1	3000	67	42	1.40%	0.80	641	339	11.30%	0.95	291	162	5.40%	0.90
2	2500	90	33	1.32%	1.36	708	339	13.56%	1.04	102	66	2.64%	0.77
3	1900	51	33	1.74%	0.77	233	146	7.68%	0.80	121	92	4.84%	0.66
4	1600	57	34	2.13%	0.84	213	129	8.06%	0.83	82	60	3.75%	0.68
5	1700	23	16	0.94%	0.72	312	175	10.29%	0.89	46	35	2.06%	0.66
6	1900	100	19	1.00%	2.63	337	172	9.05%	0.98	145	87	4.58%	0.83
7	1800	140	38	2.11%	1.84	405	160	8.89%	1.27	128	51	2.83%	1.25
8	1400	29	20	1.43%	0.73	173	115	8.21%	0.75	101	69	4.93%	0.73
9	1400	15	10	0.71%	0.75	205	117	8.36%	0.88	70	47	3.36%	0.74
10	1400	20	10	0.71%	1.00	199	130	9.29%	0.77	96	59	4.21%	0.81
11	2000	29	23	1.15%	0.63	370	227	11.35%	0.81	168	102	5.10%	0.82
12	1600	74	34	2.13%	1.09	272	169	10.56%	0.80	112	70	4.38%	0.80
13	800	9	6	0.75%	0.75	148	68	8.50%	1.09	21	16	2.00%	0.66
14	1200	32	21	1.75%	0.76	183	110	9.17%	0.83	21	16	1.33%	0.66
15	1200	25	6	0.50%	2.08	287	153	12.75%	0.94	81	48	4.00%	0.84
16	1300	16	12	0.92%	0.67	179	110	8.46%	0.81	75	52	4.00%	0.72
17	1600	46	11	0.69%	2.09	272	150	9.38%	0.91	119	90	5.63%	0.66
18	600	33	4	0.67%	4.13	155	77	12.83%	1.01	9	9	1.50%	0.50
19	1700	61	27	1.59%	1.13	519	233	13.71%	1.11	257	143	8.41%	0.90
20	900	17	11	1.22%	0.77	103	70	7.78%	0.74	36	28	3.11%	0.64
21	400	4	4	1.00%	0.50	66	37	9.25%	0.89	13	10	2.50%	0.65
22	800	12	6	0.75%	1.00	75	49	6.13%	0.77	42	31	3.88%	0.68
X	1400	6	5	0.36%	0.60	116	74	5.29%	0.78	17	15	1.07%	0.57
Y	200	0	0	0.00%	0.00	2	1	0.50%	1.00	0	0	0.00%	0.00

…

Chromosome	892				893				894				all native cells	
	Total gene mutations	Affected distinct genes	Proportion of mutated genes	Approximate mean of mutations per gene	Total gene mutations	Affected distinct genes	Proportion of mutated genes	Approximate mean of mutations per gene	Total gene mutations	Affected distinct genes	Proportion of mutated genes	Approximate mean of mutations per gene	Median of mutated genes proportions	Median of mutations per affected gene approximation
1	2089	902	30.07%	1.16	2441	995	33.17%	1.23	734	401	13.37%	0.92	**14.18%**	**0.95**
2	1407	615	24.60%	1.14	1606	663	26.52%	1.21	697	359	14.36%	0.97	**13.96%**	**1.04**
3	1293	539	28.37%	1.20	1344	551	29.00%	1.22	576	307	16.16%	0.94	**17.74%**	**0.99**
4	840	384	24.00%	1.09	928	403	25.19%	1.15	301	186	11.63%	0.81	**14.19%**	**0.96**
5	1002	412	24.24%	1.22	1119	455	26.76%	1.23	363	192	11.29%	0.95	**14.32%**	**0.94**
6	1288	483	25.42%	1.33	1627	543	28.58%	1.50	487	255	13.42%	0.95	**16.92%**	**1.23**
7	1022	411	22.83%	1.24	1225	474	26.33%	1.29	382	179	9.94%	1.07	**13.36%**	**1.25**
8	625	282	20.14%	1.11	740	314	22.43%	1.18	183	115	8.21%	0.80	**11.32%**	**0.90**
9	733	338	24.14%	1.08	956	414	29.57%	1.15	336	188	13.43%	0.89	**15.46%**	**0.93**
10	794	367	26.21%	1.08	1019	421	30.07%	1.21	373	219	15.64%	0.85	**15.14%**	**0.95**
11	1271	595	29.75%	1.07	1650	696	34.80%	1.19	414	236	11.80%	0.88	**16.73%**	**0.89**
12	1015	493	30.81%	1.03	1226	551	34.44%	1.11	518	294	18.38%	0.88	**17.53%**	**0.88**
13	359	147	18.38%	1.22	412	174	21.75%	1.18	109	71	8.88%	0.77	**10.88%**	**1.09**
14	575	271	22.58%	1.06	715	311	25.92%	1.15	274	151	12.58%	0.91	**11.25%**	**0.88**
15	607	279	23.25%	1.09	811	315	26.25%	1.29	243	147	12.25%	0.83	**15.21%**	**0.97**
16	631	296	22.77%	1.07	840	381	29.31%	1.10	239	143	11.00%	0.84	**14.92%**	**0.90**
17	1032	496	31.00%	1.04	1377	601	37.56%	1.15	362	215	13.44%	0.84	**17.88%**	**0.96**
18	379	139	23.17%	1.36	422	167	27.83%	1.26	148	85	14.17%	0.87	**15.67%**	**1.09**
19	1435	610	35.88%	1.18	2012	800	47.06%	1.26	461	247	14.53%	0.93	**23.59%**	**1.12**
20	423	219	24.33%	0.97	528	279	31.00%	0.95	207	114	12.67%	0.91	**12.17%**	**0.78**
21	194	94	23.50%	1.03	289	116	29.00%	1.25	61	40	10.00%	0.76	**13.50%**	**0.90**
22	305	160	20.00%	0.95	482	225	28.13%	1.07	91	62	7.75%	0.73	**10.75%**	**0.86**
X	495	263	18.79%	0.94	527	284	20.29%	0.93	151	94	6.71%	0.80	**8.07%**	**0.80**
Y	10	6	3.00%	0.83	7	7	3.50%	0.50	4	2	1.00%	1.00	**1.00%**	**0.79**

CARCINOMA	ROI ID	895				896				897			
Chromosome	Approximate gene number of chromosome	Total gene mutations	Affected distinct genes	Proportion of mutated genes	Approximate mean of mutations per gene	Total gene mutations	Affected distinct genes	Proportion of mutated genes	Approximate mean of mutations per gene	Total gene mutations	Affected distinct genes	Proportion of mutated genes	Approximate mean of mutations per gene
1	3000	795	419	13.97%	0.95	93	59	1.97%	0.79	63	45	1.50%	0.70
2	2500	534	289	11.56%	0.92	70	49	1.96%	0.71	134	62	2.48%	1.08
3	1900	199	125	6.58%	0.80	58	46	2.42%	0.63	63	42	2.21%	0.75
4	1600	289	159	9.94%	0.91	52	36	2.25%	0.72	37	33	2.06%	0.56
5	1700	185	110	6.47%	0.84	57	38	2.24%	0.75	23	20	1.18%	0.58
6	1900	606	256	13.47%	1.18	102	38	2.00%	1.34	102	41	2.16%	1.24
7	1800	132	81	4.50%	0.81	95	30	1.67%	1.58	47	29	1.61%	0.81
8	1400	255	142	10.14%	0.90	33	21	1.50%	0.79	29	22	1.57%	0.66
9	1400	141	90	6.43%	0.78	41	32	2.29%	0.64	30	22	1.57%	0.68
10	1400	92	66	4.71%	0.70	44	35	2.50%	0.63	37	28	2.00%	0.66
11	2000	414	238	11.90%	0.87	78	58	2.90%	0.67	86	47	2.35%	0.91
12	1600	493	253	15.81%	0.97	37	34	2.13%	0.54	49	27	1.69%	0.91
13	800	60	40	5.00%	0.75	28	21	2.63%	0.67	44	38	4.75%	0.58
14	1200	300	170	14.17%	0.88	25	17	1.42%	0.74	29	18	1.50%	0.81
15	1200	192	99	8.25%	0.97	49	19	1.58%	1.29	28	15	1.25%	0.93
16	1300	362	201	15.46%	0.90	40	24	1.85%	0.83	36	19	1.46%	0.95
17	1600	239	142	8.88%	0.84	56	40	2.50%	0.70	27	16	1.00%	0.84
18	600	26	15	2.50%	0.87	5	5	0.83%	0.50	14	7	1.17%	1.00
19	1700	704	310	18.24%	1.14	86	56	3.29%	0.77	48	31	1.82%	0.77
20	900	203	133	14.78%	0.76	26	22	2.44%	0.59	34	17	1.89%	1.00
21	400	42	30	7.50%	0.70	3	2	0.50%	0.75	0	0	0.00%	0.00
22	800	145	80	10.00%	0.91	26	12	1.50%	1.08	6	4	0.50%	0.75
X	1400	144	99	7.07%	0.73	17	14	1.00%	0.61	14	11	0.79%	0.64
Y	200	0	0	0.00%	0.00	1	1	0.50%	0.50	1	1	0.50%	0.50

…

Chromosome	904				905				906				all carcinoma cells	
	Total gene mutations	Affected distinct genes	Proportion of mutated genes	Approximate mean of mutations per gene	Total gene mutations	Affected distinct genes	Proportion of mutated genes	Approximate mean of mutations per gene	Total gene mutations	Affected distinct genes	Proportion of mutated genes	Approximate mean of mutations per gene	Median of mutated genes proportions	Median of mutations per affected gene approximation
1	1298	641	21.37%	1.01	61	49	1.63%	0.62	333	192	6.40%	0.87	**2.53%**	**0.87**
2	1050	476	19.04%	1.10	33	30	1.20%	0.55	220	147	5.88%	0.75	**2.48%**	**0.78**
3	795	397	20.89%	1.00	36	29	1.53%	0.62	186	114	6.00%	0.82	**2.47%**	**0.75**
4	548	287	17.94%	0.95	28	25	1.56%	0.56	122	74	4.63%	0.82	**2.25%**	**0.82**
5	566	292	17.18%	0.97	16	15	0.88%	0.53	162	98	5.76%	0.83	**2.24%**	**0.78**
6	840	356	18.74%	1.18	81	24	1.26%	1.69	167	108	5.68%	0.77	**2.16%**	**1.18**
7	791	336	18.67%	1.18	27	25	1.39%	0.54	222	108	6.00%	1.03	**1.67%**	**1.03**
8	331	169	12.07%	0.98	25	20	1.43%	0.63	112	68	4.86%	0.82	**1.57%**	**0.80**
9	540	263	18.79%	1.03	26	22	1.57%	0.59	121	71	5.07%	0.85	**2.29%**	**0.69**
10	596	271	19.36%	1.10	22	16	1.14%	0.69	115	84	6.00%	0.68	**2.50%**	**0.69**
11	896	437	21.85%	1.03	44	27	1.35%	0.81	203	126	6.30%	0.81	**2.90%**	**0.81**
12	648	342	21.38%	0.95	41	34	2.13%	0.60	217	138	8.63%	0.79	**2.13%**	**0.86**
13	355	146	18.25%	1.22	34	27	3.38%	0.63	76	52	6.50%	0.73	**4.75%**	**0.67**
14	355	195	16.25%	0.91	36	22	1.83%	0.82	130	77	6.42%	0.84	**3.08%**	**0.84**
15	403	200	16.67%	1.01	45	22	1.83%	1.02	74	54	4.50%	0.69	**2.17%**	**0.94**
16	430	227	17.46%	0.95	23	18	1.38%	0.64	139	88	6.77%	0.79	**1.85%**	**0.82**
17	536	297	18.56%	0.90	21	18	1.13%	0.58	152	100	6.25%	0.76	**2.50%**	**0.76**
18	139	78	13.00%	0.89	6	6	1.00%	0.50	54	39	6.50%	0.69	**1.83%**	**0.82**
19	1035	439	25.82%	1.18	23	21	1.24%	0.55	238	109	6.41%	1.09	**3.29%**	**1.04**
20	394	207	23.00%	0.95	24	21	2.33%	0.57	79	57	6.33%	0.69	**2.44%**	**0.69**
21	111	65	16.25%	0.85	4	4	1.00%	0.50	27	19	4.75%	0.71	**1.75%**	**0.71**
22	131	80	10.00%	0.82	11	8	1.00%	0.69	84	56	7.00%	0.75	**1.50%**	**0.82**
X	211	137	9.79%	0.77	14	11	0.79%	0.64	78	56	4.00%	0.70	**1.00%**	**0.73**
Y	1	1	0.50%	0.50	0	0	0.00%	0.00	0	0	0.00%	0.00	**0.00%**	**0.00**
