# Supplementary figures and images for: Enhanced Tumor Diagnostics via Cyber-Physical Workflow: Integrating Morphology, Morphometry, and Genomic MultimodalData Analysis and Visualization in Digital Pathology
Source: Sensors (Basel). 2025 Jul 17;25(14):4465. doi: 10.3390/s25144465 (PMC12300765; doi:10.3390/s25144465)

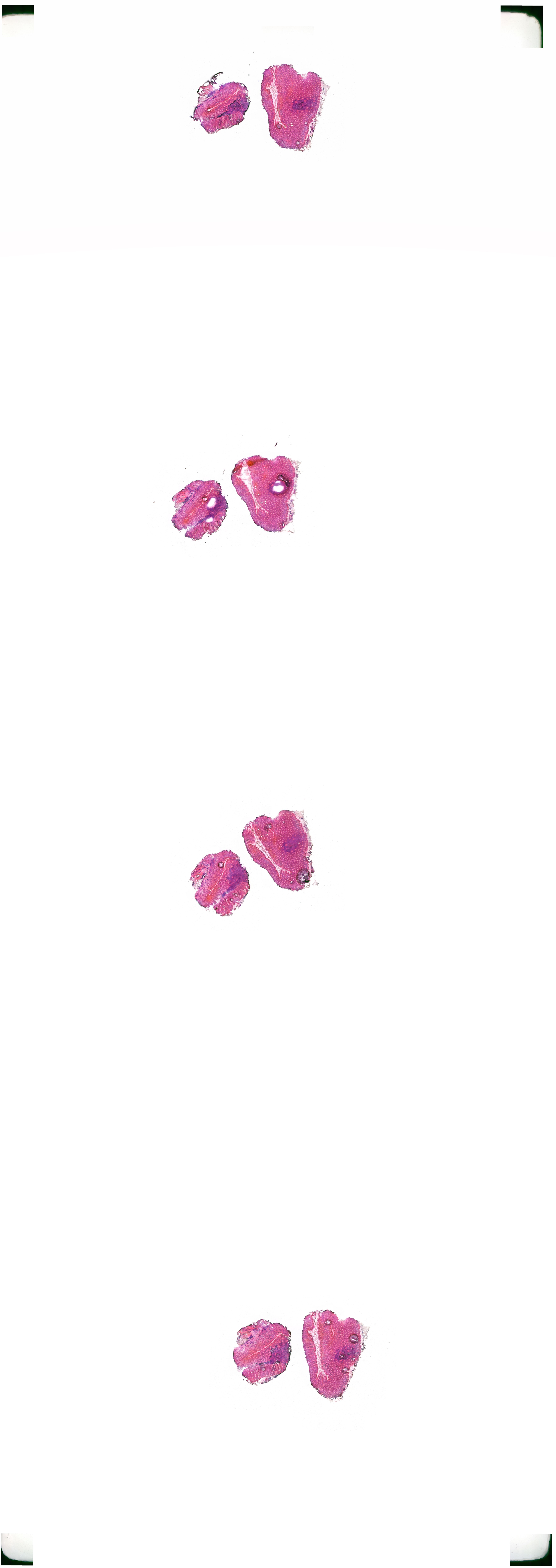

Supplement: Supplementary file 1 [file sensors-25-04465-s001.zip › S5_Normal__with_cross_cut_ref__after__40x.jpg]

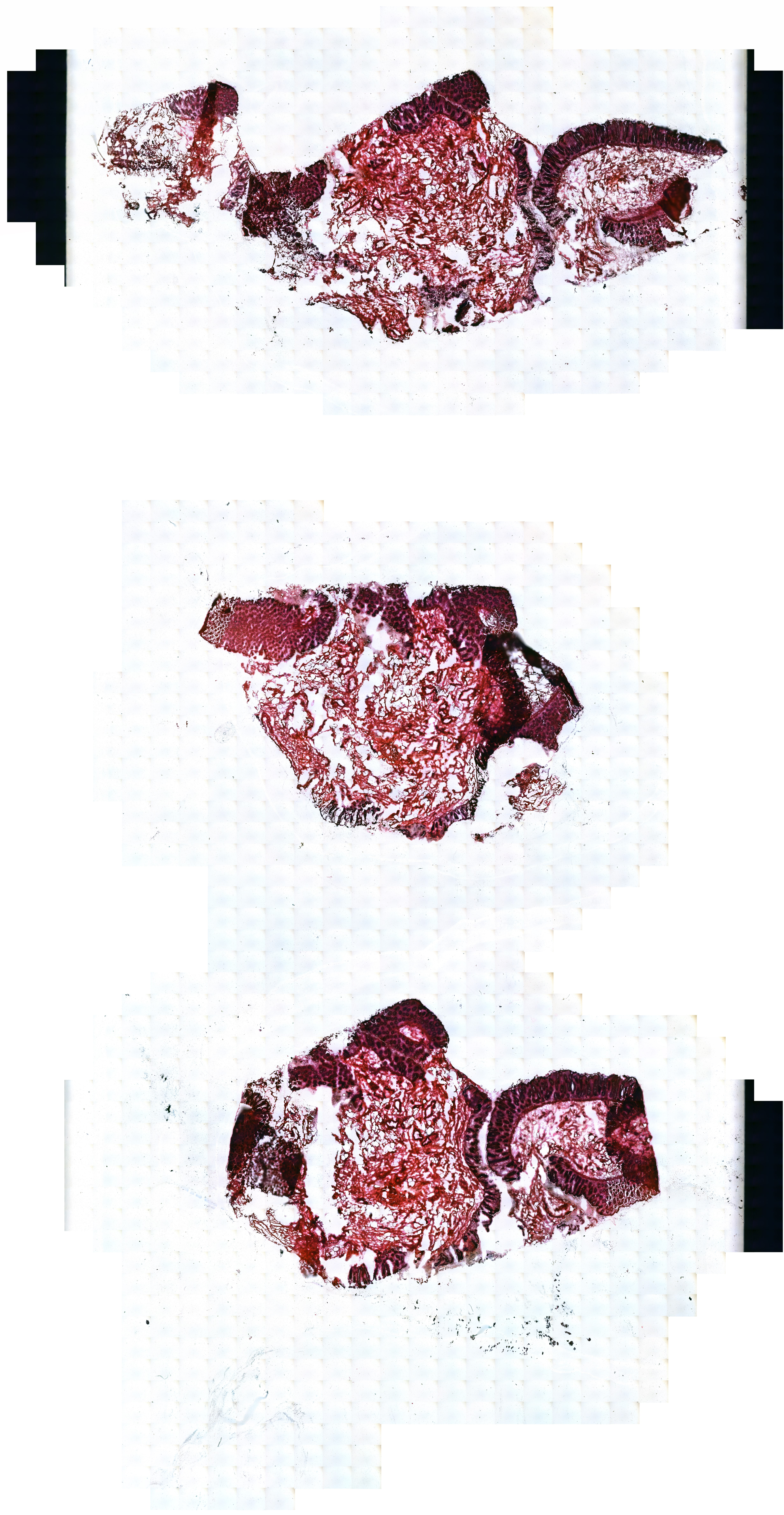

Supplement: Supplementary file 1 [file sensors-25-04465-s001.zip › S6_03_tyf_nat_16um__40x.jpg]

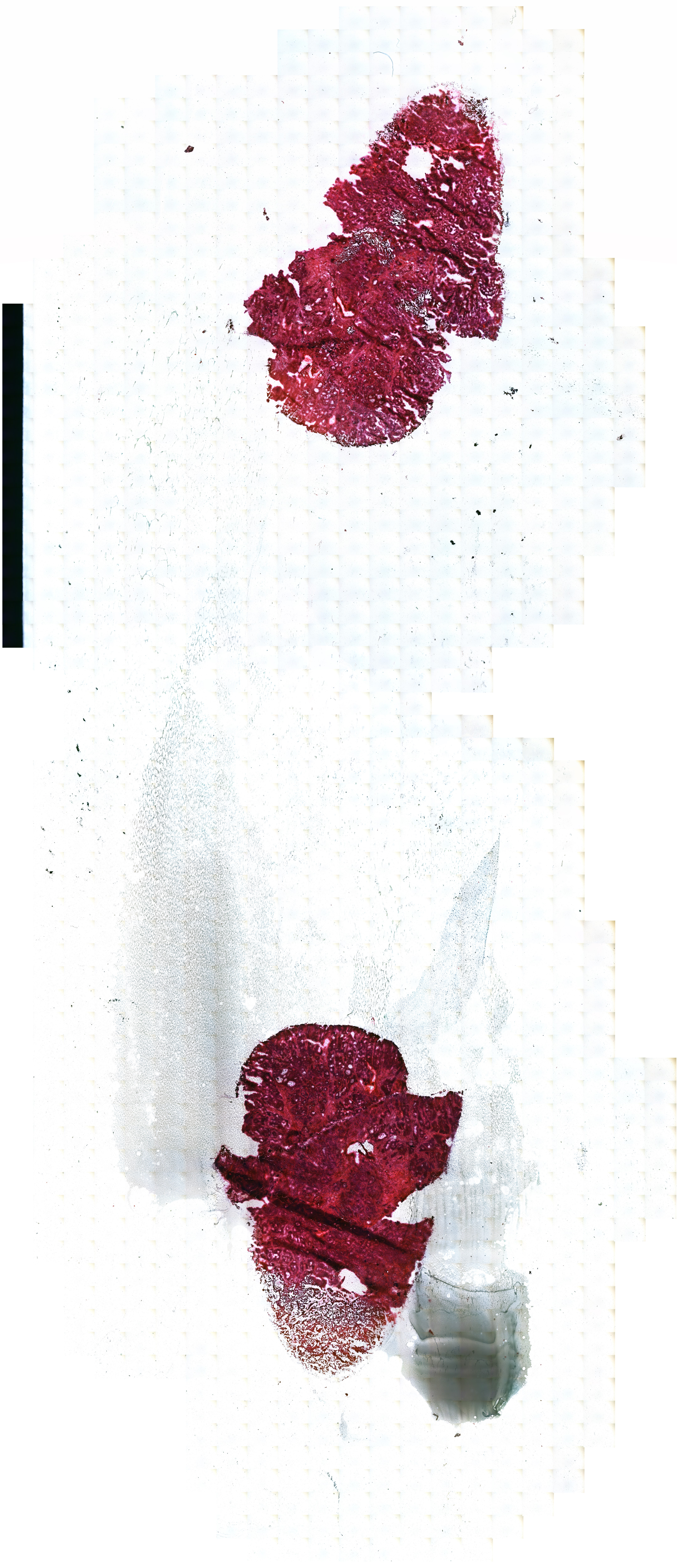

Supplement: Supplementary file 1 [file sensors-25-04465-s001.zip › S7_08_tyf_crc_16um__40x.jpg]
